# Multiple RNAs from the mouse carboxypeptidase M *locus*: functional RNAs or transcription noise?

**DOI:** 10.1186/1471-2199-10-7

**Published:** 2009-02-08

**Authors:** Alessander O Guimarães, Fabiana L Motta, Viviane S Alves, Beatriz A Castilho, João B Pesquero

**Affiliations:** 1 Departamento de Biofísica, Universidade Federal de São Paulo, São Paulo, Brazil; 2 Departamento de Microbiologia e Imunologia, Universidade Federal de São Paulo, São Paulo, Brazil

## Abstract

**Background:**

A major effort of the scientific community has been to obtain complete pictures of the genomes of many organisms. This has been accomplished mainly by annotation of structural and functional elements in the genome sequence, a process that has been centred in the gene concept and, as a consequence, biased toward protein coding sequences. Recently, the explosion of transcriptome data generated and the discovery of many functional non-protein coding RNAs have painted a more detailed and complex scenario for the genome. Here we analyzed the mouse carboxypeptidase M *locus *in this broader perspective in order to define the mouse CPM gene structure and evaluate the existence of other transcripts from the same genomic region.

**Results:**

Bioinformatic analysis of nucleotide sequences that map to the mouse CPM *locus *suggests that, in addition to the mouse CPM mRNA, it expresses at least 33 different transcripts, many of which seem to be non-coding RNAs. We randomly chose to evaluate experimentally four of these extra transcripts. They are expressed in a tissue specific manner, indicating that they are not artefacts or transcriptional noise. Furthermore, one of these four extra transcripts shows expression patterns that differed considerably from the other ones and from the mouse CPM gene, suggesting that there may be more than one transcriptional unit in this *locus*. In addition, we have confirmed the mouse CPM gene RefSeq sequence by rapid amplification of cDNA ends (RACE) and directional cloning.

**Conclusion:**

This study supports the recent view that the majority of the genome is transcribed and that many of the resulting transcripts seem to be non-coding RNAs from introns of genes or from independent transcriptional units. Although some of the information on the transcriptome of many organisms may actually be artefacts or transcriptional noise, we argue that it can be experimentally evaluated and used to find and define biological functional elements on the genome. Furthermore, the transcription of other functional RNAs besides the protein coding RNA from a specific genomic *locus *imposes extra care when designing and interpreting experiments involving genetic manipulations or expression detection and quantification.

## Background

The carboxypeptidase M (CPM) is a cell membrane metalloprotease from the CPN/E regulatory family that is expressed in varying levels in most cell types [1-3]. It is believed that this enzyme plays important roles in the processing of many peptide hormones, especially during inflammation and macrophage activation [4,5]. Both CPM activity and protein levels greatly increase in response to inflammatory stimuli [6,7]. Additionally, CPM mediated cleavage of the C-terminal arginine from kinins change their affinity to the kinin B1 and B2 receptors, and this arginine may also be important to NO production, especially in the lungs [[6,8], and [9]].

Our group, together with others, have defined experimentally the human CPM gene organization and characterized that its promoter harbours many cis-regulatory elements responsive to inflammatory stimulus [10,11]. Although the same experimental strategy has not been implemented to resolve the mouse CPM gene [GenBank – GeneID: 70574], it has been defined and annotated on the mouse chromosome 10 mainly on basis of an *in silico *analysis of the mouse transcriptome and genome using the nucleotide sequence data on the public databases as well as in comparison with the human CPM gene sequence.

This new approach to gene definition and genomic annotation is now feasible due to the explosion of nucleotide sequence data generated by many groups, including genome and transcriptome projects. However, most of these genome annotations and transcriptome analysis have focused on protein coding sequences and have, until recently, greatly disregarded the amount of non-protein-coding nucleotide sequences transcribed by the genomes of eukaryotes [12-14]. Non-protein-coding RNAs seem to account for a great portion of the transcriptome, for example: 34,030 of the 102,281 mouse cDNAs generated by the FANTOM3 project lack any protein-coding sequence (CDS) and are annotated as non-protein coding RNA (ncRNA) [15]; and more than half of the detected transcribed sequences from the human genome regions analyzed by the ENCODE project are not observed to align with their annotated protein coding genes [16]. Taking into consideration this new genome scenario, and in order to validate the mouse genome annotation for the CPM gene, we analysed here a number of nucleotide sequences from various public data banks that map to the mouse CPM genomic region. These sequences, which include ESTs and mRNAs of mouse CPM GenBank-UniGene entry Mm.339332 and cap analysis of gene expression-tags (CAGE-tags) [17-20] are presented as supporting evidence for this gene annotation and definition, although not all of these cDNAs and CAGE-tags have exactly the same nucleotide sequence as the proposed CPM gene. In fact, some of these mouse CPM supporting sequences in the databases have completely different nucleotide sequences. Therefore, we focused in defining all possible extra transcripts of the mouse CPM *locus *on the basis of these cDNAs and CAGE-tags and their annotation to the mouse CPM genomic *locus*. We also evaluated experimentally the mouse CPM gene structure and the expression of some of these proposed extra transcripts from mouse CPM *locus*, in addition to the CPM gene in selected mouse tissues.

## Results and discussion

### Bioinformatic analysis of the mouse CPM *locus *and its transcripts

**Figure 1:**
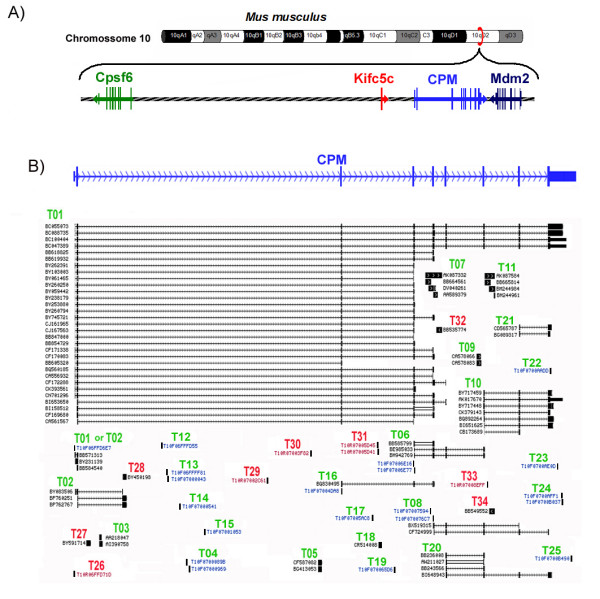
**The mouse CPM *locus *and its transcripts**. A) The genomic region on mouse chromosome 10 where the CPM gene maps is represented showing the relative position and direction of transcription of the Cpsf6 (in green), CPM (in blue) and Mdm2 (in dark blue) genes as well as the pseudogene Kifc5c (in red). B) The analyzed cDNAs and CAGE sequences that support the defined mouse CPM *locus *transcripts are shown in relation to the mouse CPM gene exons. The black vertical lines and blocks represent the nucleotide sequences of the cDNA or CAGE-tag(s) in their relative position to the mouse CPM gene exons (represented in blue), identified by the cDNA GenBank Accession number (in black) or the CAGE transcription Star Site ID (CAGEtss, in blue). The exons of spliced cDNAs are connected by horizontal arrowed lines showing the direction of transcription. The mouse CPM *locus *transcript(s) being supported by these sequences are indicated above the cDNAs and CAGEtss codes as "T" and the assigned number, in green when sense, and in red when anti-sense to the CPM gene direction of transcription.

The mouse CPM *locus *is located in the 10D2 region of the mouse chromosome 10, between the Mdm2 gene (transformed mouse 3T3 cell double minute 2) [GenBank – Gene ID: 17246] and the annotated mRNA for kinesin-related protein KIFC5C [GenBank: AF221104], which is a possible pseudogene of the kinesin family member C1 [GenBank – GeneID: 16580] that maps to the intergenic region between the Cpsf6 (cleavage and polyadenylation specific factor 6) [GenBank – GeneID: 432508] and CPM gene at about 35 kb upstream from the 5'end of the CPM gene (Figure 1A). We first designated the mouse CPM gene RefSeq sequence [GenBank: NM_027468] as the mouse CPM *locus *Transcript 01 (mCPM-T01). This 5195 nucleotides long sequence is distributed in 9 exons in a genomic region of about 56 kilobases [21].

In addition to the CPM gene (mCPM-T01), we defined at least 33 other possible mouse CPM *locus *Transcripts (mCPM-Ts) after analysing a total of 237 sequences that maps to this *locus *(Figure 1A): 38 CAGE-tags (see Additional file 1) and 199 cDNA sequences between mRNAs and ESTs from the mouse CPM GenBank – UniGene [22] entry Mm.339332 and other cDNA sequences deposited in the GenBank (see Additional file 2). Sixteen of these 33 possible mCPM-Ts were defined based only on CAGE-tag sequences (table 1), and all the information we have about them is the 18 to 20 initial nucleotides, the direction of their transcription and the source samples [17-20]. The other 17 mCPM-Ts were defined based on at least one cDNA sequence, and seven of them are based on cDNAs of spliced RNAs (mCPM-Ts: 02, 06, 08, 10, 16, 20 and 22). Except for the mCPM T02 and mCPM-T10, all the other five spliced mCPM-Ts are totally contained in the mCPM-T01 sequence (Table 1), and may be incomplete cDNAs from the mCPM-T01. However, three of these five mCPM-Ts (mCPM-T06, mCPM-T08 and mCPMT16) have CAGE-tags that map at their beginnings, supporting their definition as independent transcripts (Figure 1 and table 1). The mouse CPM-T02 shares with mCPM-T01 its two initial exons but ends on a completely different third exon located on the second intron of the CPM gene. Thus it is very likely that its transcription is under control of the mouse CPM gene promoter. The mCPM-T10 transcript, on the other hand, shares the last 3 exons of mCPM-T01, but it begins at 48 nucleotides upstream of the mouse CPM gene exon 7 (Figure 1). Furthermore, we have evidence that its expression is controlled by a promoter region different from that of the mouse CPM gene, as discussed later.

**Table 1:** Mouse CPM *locus *transcripts defined by bioinformatics analysis

		**Number of supporting sequences**					**CPC**	
**Transcript**	**DNA strand**	**Total**	**Unique** **cDNA**	**Spliced** **cDNA**	**CAGE**	**Size** **(bp)**	**coding potential**	**score**
**01**	Sense	53^#^	31	52	3^¥^	5195	Strong	6.01
**02**	Sense	9	3	3	3^¥^	798	Weak	0.48
**03**	Sense	2	2	0	0	391	No	-1.28
**04**	Sense	7	0	?	7	?	?	?
**05**	Sense	2	2	0	0	408	No	-1.09
06 *	Sense	6	0	3	3	652	Strong	3.70
**07**	Sense	4	4	0	0	1900	Weak	0.37
08 *	Sense	5	0	2	3	1014	Strong	3.52
**09**	Sense	2	2	0	0	548	No	-1.05
**10**	Sense	7	3	7	0	2007	Strong	1.73
**11**	Sense	4	4	0	0	1125	No	-1.15
**12**	Sense	1	0	?	1	?	?	?
**13**	Sense	2	0	?	2	?	?	?
**14**	Sense	1	0	?	1	?	?	?
**15**	Sense	1	0	?	1	?	?	?
16 *	Sense	2	0	1	1	502	Strong	2.68
**17**	Sense	2	0	?	2	?	?	?
**18**	Sense	1	1	0	0	332	No	-1.35
**19**	Sense	1	0	?	1	?	?	?
20 *	Sense	4	0	4	0	890	Strong	2.79
21 *	Sense	2	0	2	0	547	Strong	1.84
22 *	Sense	1	0	?	1	?	?	?
23 *	Sense	1	0	?	1	?	?	?
24 *	Sense	2	0	?	2	?	?	?
25 *	Sense	1	0	?	1	?	?	?
26 *	Anti-sense	1	0	?	1	?	?	?
**27**	Anti-sense	1	1	0	0	492	Weak	0.02
**28**	Anti-sense	1	1	0	0	468	No	-1.11
**29**	Anti-sense	1	0	?	1	?	?	?
**30**	Anti-sense	1	0	?	1	?	?	?
**31**	Anti-sense	4	0	?	4	?	?	?
**32**	Anti-sense	1	1	0	0	662	No	-1.27
**33**	Anti-sense	1	0	?	1	?	?	?
**34**	Anti-sense	1	1	0	0	668	No	-1.28
**Undefined**^&^	Both	124	124	0	5	N/A	N/A	N/A

* Transcripts totally included in the mCPM-T01.

^&^Non-spliced cDNAs that map in the last exon of mouse CPM gene and probably represent independent transcripts.

^#^Not including the cDNAs that map to the last exon.

^¥ ^Can be supportive of mCPM-T01 or mCPM-T02.

? = Not enough information to conclude. N/A = not applicable.

The other 10 mCPM-Ts defined here on cDNA basis were all defined from the sequence of non-spliced cDNAs that map to the mouse CPM *locus*, and half of them have only one cDNA sequence that supports their definition (a so called non-spliced "singleton" cDNA). These non-spliced singleton sequences are very abundant in many cDNA databanks and have a high chance of being artefacts such as DNA contamination, unprocessed pre-mRNA, cloned spliced-out intronic sequences or simply mistakes of the transcription machinery (transcription noise) [23]. Four of these five transcripts defined on the basis of only a singleton non-spliced cDNA mapped to the anti-sense DNA strand of the CPM gene (Table 1).

Most of these 33 mouse CPM *locus *transcripts defined here seem to be non-coding RNAs. Except for mCPM-T02, that received a weak coding potential score, all of the spliced mCPM *locus *transcripts scored high on the protein coding analysis using the CPC program (table 1) [24]. However, the high score for the majority of these transcripts may actually reflect the fact that most of their sequence is contained in the mouse CPM gene. In fact, the CPC program takes into account in the calculation of the coding potential the similarity with known protein sequences deposited in the UniRef90 databank [24], which includes the mouse CPM sequence. On the other hand, all the non-spliced mouse CPM *locus *transcripts scored very low in the CPC program which classified them as with no coding potential, except for mCPM-T07 and mCPM-T27, which received a weak protein coding potential (table 11). In the case of mCPM-T07 this weak coding potential may also be reasoned because it contains part of the coding sequence of the CPM gene. As expected, the highest coding potential score was that of the mCPM-T01, the mouse CPM gene (table 1).

Finally, there are 124 cDNAs of non-spliced RNAs that map exclusively to the exon 9 of the CPM gene, supporting the existence of other mouse CPM *locus *transcripts besides the 33 defined above. However, because we cannot rule out that they are incomplete cDNAs from some of the already defined mCPM-Ts, we defined only 4 transcripts there based on 5 CAGE-tags that map to this genomic region (Figure 1, mCPM-Ts: 22 to 25). Furthermore, it is still very intriguing that several independent, mainly non-coding, RNA transcripts come from the sequences of the last exon of the genes [25,26]. This seems to be very frequent in genes in the so called genomic head to tail orientation, where a gene is in close proximity to opposite DNA strand neighbours, and these last exons transcripts are suggested to play regulatory roles [15,27].

### Experimental analysis of the mouse CPM locus transcript 01, the mouse CPM gene

**Figure 2:**
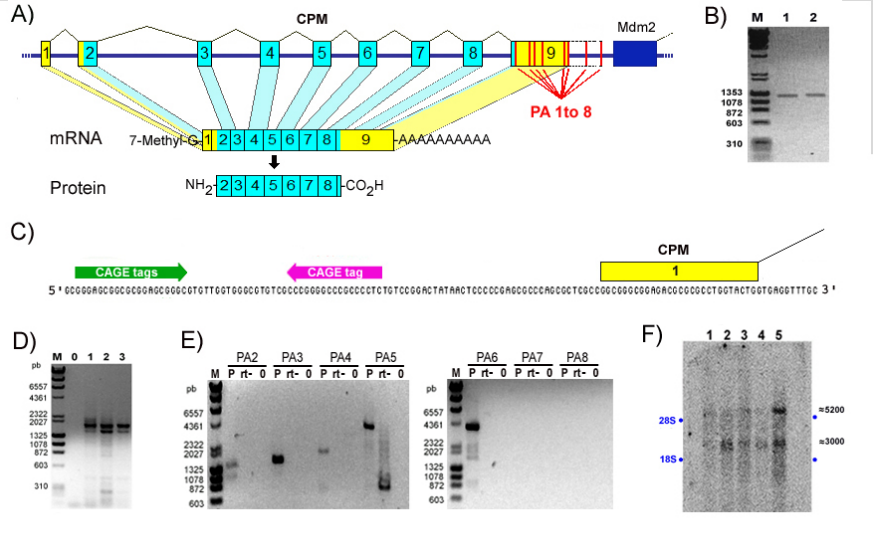
**The mouse CPM gene**. (A) Representation of the mouse CPM gene, mRNA and protein showing the exons as numbered blocks, the untranslated and the coding regions in yellow and blue respectively, and the poly-A signals (PA1 to 8) as red lines. The dark blue block represents the Mdm2 gene last exon. (B) The cDNA fragments for the mouse CPM gene coding region obtained in two independent RT-PCRs. (C) Genomic DNA sequence of the 5'end of the mouse CPM gene indicating the positions of the CPM exon 1 (yellow block), three sense CAGE-tags (green arrow) and one anti-sense CAGE-tag (red arrow). (D) The 3'RACE PCRs from: adult mouse lung (1), 14 day embryo (2), 14 day placenta (3) and no template as negative control (0). (E) Long range RT-PCRs to evaluate the use of mouse CPM Poly-A signals 2 to 8. (F) Mouse CPM specific northern blot of total RNA from: 14 day embryo RNA (1), 14 day placenta RNA (2), 14 day embryo red organs (3), 18 day placenta (4) and 18 day embryo red organs (5). The position of the ribosomal RNAs 28S and 18S are indicated as blue dots, and the approximated size of the visualized bands in number of nucleotides are shown at right in black. The RT-PCRs and 3'RACE results shown here are negative images of UV light visualized 1% agarose gel electrophoresis stained with ethidium bromide, and M indicates the DNA size standards with some of their DNA fragments size shown as base pairs.

From the 237 sequences analysed, there are 31 cDNAs whose sequences are totally included in the mouse CPM RefSeq nucleotides and therefore cannot be attributed to any other transcript in the *locus *besides the mCPM-T01 (table 1). The mCPM-T01 sequence coding for mouse CPM protein, a 443 amino acids polypeptide that shares about 84% similarity with the human CPM protein, begins in the second exon and extends to the beginning of the last exon – the exon 9 (Figure 2A). This mCPM-T01 coding region was confirmed by RT-PCR, cloning and sequencing (Figure 2B).

We also obtained for the start of transcription of mCPM-T01 the same initial nucleotides of the mouse CPM gene RefSeq sequence in RACE 5'experiments using an anti-sense primer directed to the beginning of the mouse CPM exon 3 (Figure 2C and Additional file 3), besides confirming the splicing junctions of exons 1, 2 and 3. These 5' RACE experiments did not confirm a possible start of transcription 74 nucleotides upstream of this point, supported by 3 CAGE-tags in the sense orientation [CAGE TSS ID: T10F06FFD6E7] (Figure 2C). Furthermore, none of the 199 cDNAs analysed here were found to begin upstream of the 5'RACE confirmed start site. Out of the 30 cDNAs that map their beginning to the initial region of the CPM gene, 16 share the same start as the mouse CPM RefSeq sequence and the other 14 begin 1 to 15 nucleotides downstream of this start site (see Additional file 4). However, we cannot rule out the possibility that these 3 CAGE-tags that map upstream of the mCPM-T01 start may actually be the beginning of the mCPM-T02 transcript, or that they are tissue specific, since we did not test the same tissues that originated these CAGE-tags nor performed 5'RACE experiments with mCPM-T02 specific primer.

There are eight classical poly-A signals located at the 3'end of the mouse CPM *locus *that could be used to process the 3'end of the mCPM-T01 mRNA (genomic sequence AATAAA), which we designated poly-A signal 1 to 8 (PA 1 to 8) (figure 2A and Additional file 5). This genomic region extends from the mouse CPM RefSeq last exon into the intergenic region between the end of this exon and the end of the Mdm2 gene last exon. We could not find any individual cDNA on the GenBank [28] that extended through the entire sequence of the mouse CPM RefSeq or that extended further than the third poly-A signal cleavage site when having all the exons. This can be easily explained by the existence of adenosine rich stretches in the last exon of the mouse CPM RefSeq that could function as an internal annealing site to the oligo dTs used in the construction of the cDNA libraries [23](see Additional file 5).

Analysis by 3'RACE experiments confirmed the use of the third classical poly-A signal in this region (PA3), located at nucleotide 2723 of the mouse CPM RefSeq sequence [GenBank: NM_027468] (Figure 2D). We also obtained in the 3'RACE experiments one clone of the 3'end of the possible CPM mRNA that ended exactly after the fourth poly-adenosine signal (PA4) in a stretch of poly-adenosines also present in the corresponding genomic sequence beginning at nucleotide 3146 of the CPM RefSeq sequence. Therefore, we cannot exclude that this clone was the result of internal annealing of the oligo dT 3'RACE adapter to longer mRNAs (Figure 2D and Additional file 5). In addition, we obtained by long-range RT-PCR cDNA fragments that supported the use of poly-A signals 4, 5 and 6, but we were unable to obtain fragments that evidenced the use of the two most downstream poly-A signals in the 3'end of the mouse CPM *locus*, PA7 and 8 (Figure 2E).

Northern blot analysis of total RNA from 5 different mouse samples and a probe that anneals to the exons 8 and 9 of the mouse CPM mRNA detected three major RNAs that appear to be 2.8 kb, 3.0 kb and 5.2 kb long (Figure 2F), which are in accordance with the use of poly-A signals 3, 4, 5 or 6 with the addition of a 80 to 90 nucleotide long mature poly-A tail [29,30].

Together, these results confirm the RefSeq sequence of the mouse CPM as the longest possible mouse CPM *locus *transcript, the mCPM-T01, but also indicate that at least two smaller mRNAs for the mouse CPM protein are transcribed from the mouse CPM gene varying mainly in the size of their 3'untranslated end.

### Detecting other mouse CPM *locus *transcripts in mouse tissues

**Figure 3:**
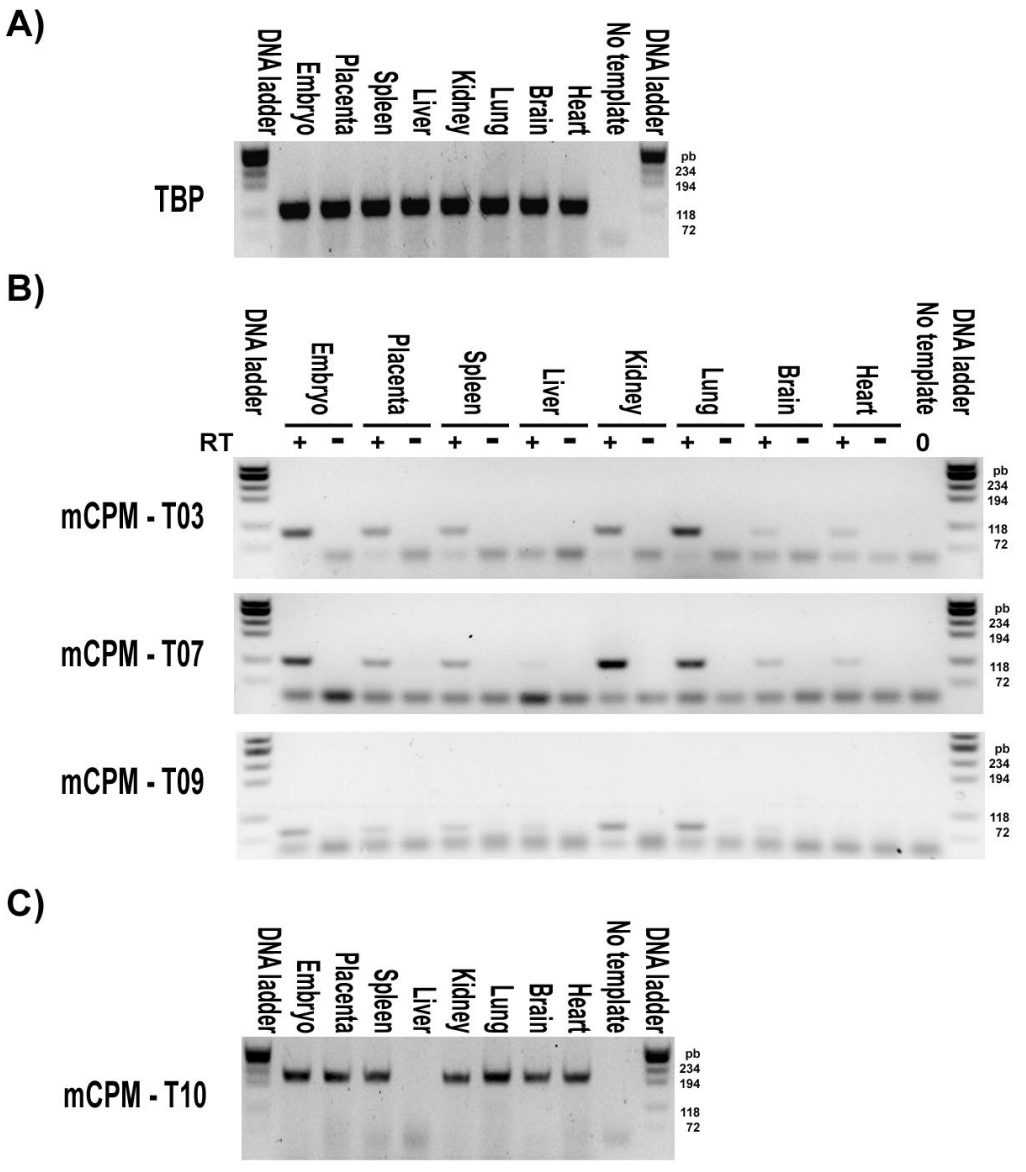
**Detecting other mouse CPM *locus *transcripts**. Negative images of 2% agarose gels stained with ethidium bromide and visualized under UV light after electrophoresis of the following reactions: (A) PCRs confirming the good quality of the reversed transcribed RNAs of the indicated mouse samples by detecting the expected TBP gene transcript fragment. (B) PCRs using as template the same reversed transcribed RNAs of the indicated mouse samples used in the previously panel (RT+) or their no reverse transcriptase control reactions (RT-) to detect the mouse CPM *locus *transcripts 03, 07 and 09. (C) PCRs to detect the mouse CPM *locus *transcript 10 in the reversed transcribed RNAs of the indicated mouse samples. No template PCRs for all experiments are indicated. The DNA ladder marker ϕX174RF DNA/Hae III fragments (Invitrogen) was used in all gels and the size of the lowest four bands in base pairs are indicated. Control PCRs using as template the respective no reverse transcriptase reaction for each mouse sample (RT-) were performed to confirm no amplification from genomic DNA contamination only for the mCPM-T03, 07 and 09 because designed primers anneal to the same exon of the targeted transcripts.

We randomly chose to check by RT-PCR the existence of RNAs from four of the possible mouse CPM *locus *transcripts. We defined these *locus *transcripts by bioinformatics analysis based on more than one cDNA sequence, and they are: mCPM-T03, mCPM-T07, mCPM-T09 and mCPM-T10. We were able to amplify by RT-PCRs the expected cDNA fragments from all these four possible mouse CPM *locus *transcripts in almost all tested mouse samples, confirming their existence (Figure 3). We also confirmed the sequence of the RT-PCR amplicons by cloning and sequencing the DNA fragments of the expected sizes obtained in previously performed experiments (data not shown).

### Quantitative RT-PCR analysis in mouse tissues of five CPM *locus *Transcripts

**Figure 4:**
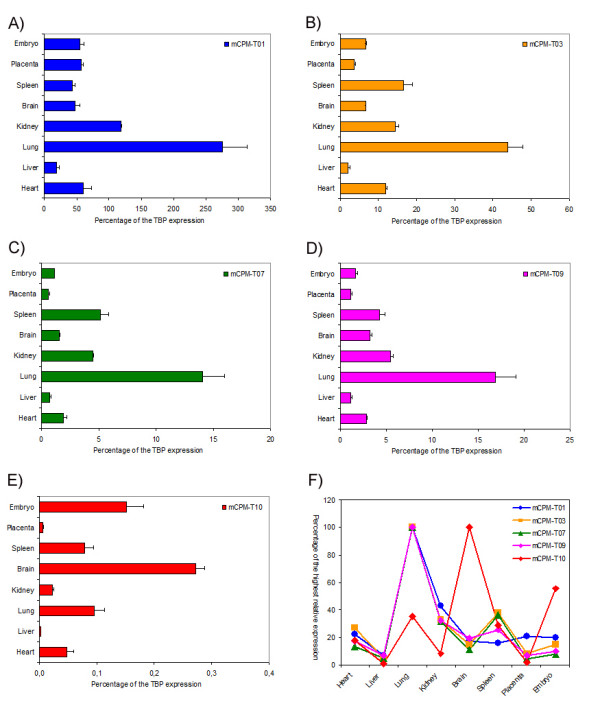
**Expression analysis of some mouse CPM *locus *transcripts**. The level of expression in mouse organs and embryos of five mouse CPM *locus *transcripts (mCPM-Ts) is shown in panels A to E. The expression levels of mCPM-Ts were calculated as the mean relative expression value of three independent samples and presented as percentage of the respective mean TBP gene expression on these three samples. A comparison of the analysed mCPM-Ts pattern of expression in the indicated mouse organs and embryo is presented as a percentage of the highest relative expression in panel F.

To validate the expression of mouse CPM *locus *transcripts 03, 07, 09 and 10 and rule out that they were the result of transcription noise we analysed the level of expression by quantitative RT-PCR of each of these mCPM-Ts and the mouse CPM gene (mCPM-T01) relative to the expression of the housekeeping gene TBP in three independent samples of 6 mouse adult organs, in 14 day embryos and placentas (Figure 4 and table 2).

**Table 2:** The relative expression of some of the mouse CPM *locus *transcripts

	mCPM-T01		mCPM-T03		mCPM-T07		mCPM-T09		mCPM-T10	
**Sample**	Mean	CV (%)	Mean	CV (%)	Mean	CV (%)	Mean	CV (%)	Mean	CV (%)
**Heart**	61.2	32.6	11.8	7.0	1.9	24.1	2.9	2.8	0.048	39.8
**Liver**	18.1	55.7	2.1	34.4	0.7	26.8	1.2	17.4	0.002	39.1
**Lung**	275.2	24.6	43.9	15.3	14.1	23.3	16.9	23.3	0.096	33.5
**Kidney**	117.9	2.0	14.5	10.3	4.5	1.6	5.5	9.3	0.022	12.4
**Brain**	47.8	24.0	6.7	3.2	1.6	5.4	3.3	12.2	0.272	10.2
**Spleen**	43.4	17.8	16.6	24.4	5.1	26.5	4.3	22.4	0.079	34.8
**Placenta**	57.5	10.3	3.7	20.1	0.6	17.6	1.1	23.8	0.005	47.0
**Embryo**	54.5	21.8	6.6	11.2	1.1	4.3	1.7	17.7	0.151	34.4

The mean relative expression evaluated by qRT-PCR of the indicated mouse CPM *locus *transcripts in different mouse organs and 14 day gestation Embryo, expressed as percentage of the respective mean TBP gene expression. All values are calculated from individual values of relative expression of three animals for each sample and their coefficient of variation (CV) is presented as percentage of the mean value.

As expected, the highest expression detected was that of the mouse CPM gene (mCPM-T01) in lungs, where it was almost three times the TBP expression (Figure 4A and table 2).

The mCPM-T01 relative expression in any particular tissue or in the embryos or placentas did not differ in each of the three independent samples of the same kind, as shown by the low coefficient of variation (CV) of the mean relative expression calculated from the results of three independent samples (Table 2). This was also the case for the other four mouse CPM *locus *transcripts quantified, demonstrating that they possess a tissue specific level of expression that does not vary between similar individuals (Table 2). These results indicate that these extra four CPM *locus *transcripts are not the result of transcriptional noise, since one would expect values of expression with greater variability even between the same types of samples of different animals because of the random nature of such phenomenon.

The mCPM-T03, mCPM-T07 and mCPM-T09 patterns of expression in the various tissues were very similar to that of the CPM gene (with p < 0.001 significant Pearson correlation coefficients of respectively: 0.92, 0.93 and 0.96), which suggests that they may be part of the same transcriptional unit, and therefore be under the same transcriptional regulatory control. The only small divergence from the mouse CPM gene expression was that the expression of these mCPM-Ts in spleen was comparable to their expression in kidney (Figure 4F).

The mCPM-T03 and 09 map to intronic regions of the mouse CPM gene, and may well be processed spliced-out intronic RNAs from the mouse CPM pre-mRNA, which could explain the same expression pattern. These transcripts may still be functional non-coding RNAs under the same transcriptional control as the CPM gene and part of the regulatory RNA network [13,14].

On the other hand, the similarity of the expression patterns of mCPM-T07 and the mouse CPM gene cannot be explained in the same way as the patterns of mCPM-T03 and mCPM-T09 expressions, since this non-spliced transcript includes in its sequence the entire mouse CPM gene exon 5 and extends at least for other 800 nucleotides in both introns 4 and 5 (see Additional file 6). However, we cannot rule out the possibility that the mCPM-T07 measured expression is in reality detection of the mouse CPM pre-mRNA. Further investigation is needed to exclude this possibility and validate mCPM-T07 as a real transcript.

The mCPM-T10 is the only of the four extra mouse CPM *locus *transcripts evaluated that is processed into a spliced RNA. It is expressed in all samples tested in a much lower level than the mouse CPM gene (mCPM-T01) or the other three extra transcripts evaluated (Figure 4E and Table 2). Additionally, the mCPM-T10 presented an expression pattern that differed considerably from the other mCPM-Ts evaluated and from the mouse CPM gene (Figure 4F), with no significant correlation (p > 0.5 for all comparisons, and coefficients of correlation with the expression pattern of the other analysed mCPM-Ts ranging from -0.02 to 0.08). This indicates an independent control of transcription from the mouse CPM gene for this transcript, and suggests the existence of other transcriptional units in the *locus*.

It is also very interesting that the bioinformatic defined sequence of the mCPM-T10 transcript carries a coding region for a protein with 195 amino acids that differs only in its seven initial N-terminal amino acids from the C terminal sequence of the mouse CPM protein. This hypothetical protein, if translated from the mCPM-T10 RNA, would include the C terminal glycosylphosphatidylinositol anchor (GPI anchor) site and part of the catalytic domain, which would very unlikely fold in a similar way as in the full length mouse CPM protein. Whether mCPM-T10 is an authentic coding or non-coding transcript still awaits further investigation.

## Conclusion

These results support the recent view that the majority of the genome sequence is transcribed, and that many of the resulting transcripts seem to be non-coding RNAs exerting many different cellular functions. The existence of these extra transcripts can be deduced from the information on the transcriptomes deposited in the various public data banks, even though part of this information may actually be artefacts or transcriptional noise. However, we argue that we can still reliably use this information to find and define biological functional elements on the genome in addition to protein coding genes, but they have to be carefully experimentally validated.

The existence of multiple transcripts from the same transcriptional unit greatly enhances the possibility of genetic interactions between different genomic *loci *and gives support to the notion of a regulatory network based on RNAs [13,14]. Furthermore, our results also support the view that a specific genomic region may harbour independent transcriptional units.

The existence of both, unknown transcripts being made from a transcriptional unit of a *locus *or unknown independent transcriptional units in the same *locus*, is especially important in interpreting the results of genetic manipulations such as transgenic and knockout models as well as genetic screening studies, since the observed phenotype variation may actually be resulting from unpredicted disturbances on them. And finally, expression detection and quantification studies have to take into account the existence of extra transcripts in the same target region when designing the experiments, at the risk of obtaining misleading measurements.

## Methods

### Bioinformatic analysis

We defined the possible mouse CPM *locus *transcripts (mCPM-Ts) by analysing the cDNAs and CAGE-tag sequences that mapped to the genomic sequence *locus *using the Blat and genome browser tools of the USCS Genome Browser Database [31-33] as well as the BlastN tools of the NCBI [22,34]. The coding potential of the defined mCPM-Ts was evaluated using the CPC program [24]. The comparison between the mouse and human CPM protein was made using the BlastP tool on the NCBI [22,34].

### Animals and sampling

All animals used in this study were C57Bl/6 mice 3 to 4 month old from the Centro de Desenvolvimento de Modelos Experimentais para a Medicina e Biologia of the Federal University of São Paulo, where they were maintained on standard mouse chow at 22°C on 12 h light-dark cycle allowed *ad libitum *access to food and tap water. All experiments reported have been conducted as stated in the NIH guide for the care and use of laboratory animals [35] and approved by a local animal care and use committee [Protocol: 2007111494137]. Embryos and placentas were collected from 8, 14 and 18 day pregnant female mice, and all the other tissues from male mice. Mouse resident peritoneal cells (MPCs) were obtained by centrifugation for 3 minutes at 300 g of a 2 ml per animal of cold PBS injected intraperitoneally and subsequently collected from the peritoneal cavity of four CO_2 _sacrificed males. The samples were either immediately processed to obtain the total RNA in TRIzol reagent or frozen in liquid nitrogen and stored at -80°C for future use.

### RNA extraction

Total RNA from samples collected in TRIzol reagent were isolated as instructed by the manufacturer, and resuspended in Milli-Q academic system (Millipore) purified water. The RNAs concentrations were measured with the NanoDrop 1000 (Thermo scientific) instrument and their integrity was confirmed by visualizing under a UV illumination the rRNAs bands after electrophoresis of 1 μg of total RNA/per sample in a 1% agarose Gel stained with ethidium bromide.

### RT-PCR and Long Range RT-PCR

We obtained total cDNA for each sample with the M-MLV Reverse Transcriptase (Invitrogen) from 5 μg of the respective purified total RNA after treating it with RQ1 RNAse-Free DNAse (Promega), using these products suggested protocols. We also obtained a negative control reverse transcription reaction in parallel for each sample using the same protocol above but omitting the reverse transcriptase in order to test for genomic DNA contamination on the total RNAs. For each RT subsequent PCR we used 5 μl of a 1/50 diluted total cDNA or respective negative control reverse transcriptase reaction as template in a 50 μl final volume reaction as follows.

i) To obtain the two mouse CPM coding cDNA fragments of figure 2B we used an equal amount mixture of the 1/50 diluted lung total cDNAs from 3 animals as template, the forward and reverse primers pmCPM2f2 + pmCPM9r3 for reaction 1 and pmCPM2f3 + pmCPM9r2 for reaction 2, and the Platinum Taq DNA Polymerase High Fidelity (Invitrogen) as suggested by the manufacturer with the temperature protocol: 94°C for 3 min, 35 cycles of a denaturing step of 94°C for 15 seconds, a primer annealing step of 60°C for 30 seconds and an extension step of 68°C for 1.5 minutes.

ii) The long range RT-PCR reactions were performed with the Elongase Amplification System (Invitrogen) as suggested by the manufacturer, using a similar temperature protocol as above, except that the extension step duration varied depending on the reverse primer used with the forward primer pmCPMe8f, as follows: pmCPMpa2 for 1.5 minutes, pmCPMpa3 for 2 minutes, pmCPMpa4 for 2.5 minutes, pmCPMpa5 for 5 minutes, pmCPMpa6 for 5 minutes, pmCPMpa7 for 7 minutes and pmCPMpa8 for 7 minutes. as templates either: The same lung total cDNA, the respective control reverse transcriptase reaction (without the enzyme) and Milli-Q purified water was used as templates for the PCRs as indicated in figure 2D.

iii) To detect by RT-PCR the mCPM-Ts and the TBP expression shown on figure 3 we used the TAQ DNA polymerase (Invitrogen) as suggested by the manufacturer with cycling temperatures protocol of: 94°C for 3 min; 3 cycles of 94°C for 15 seconds and 72°C for 30 seconds; 3 cycles of 94°C for 15 seconds, 65°C for 15 seconds and 72°C for 30 seconds; 3 cycles of 94°C for 15 seconds, 63°C for 15 seconds and 72°C for 30 seconds; 30 cycles of 94°C for 15 seconds, 60°C for 30 seconds and 72°C for 30 seconds. We also used as templates for these PCRs 1/50 diluted total cDNA of each indicated sample and as negative control the respective negative control reverse transcription reaction (indicated as RT-); and the forward and reverse primers pairs: pTBPf+ pTBPr for the TBP gene, pmCPMt03f + pmCPMt03r for mCPM-T03, pmCPMt07f + pmCPMt07r for mCPM-T07, pmCPMt09f + pmCPMt09r for mCPM-T09 and pmCPMt10f + pmCPMt10r for mCPM-T10. We also performed PCRs with no template for all the primer sets. Because the fragments amplified by the TBP gene and mCPM-T10 primers pairs from cDNA extends through exon-exon junctions, only the no template PCRs were performed as control. The sequence of all oligonucleotides used as primers are presented in Additional file 7.

The DNA ladder markers ϕX174RF DNA/Hae III fragments and λDNA/Hind III fragments (Invitrogen) where used, either together or separately, as DNA size standards in all agarose gel electrophoresis performed.

### Cloning and sequencing

All the RT-PCR DNA fragments that were cloned here were first collected from ethidium bromide stained agarose gel after electrophoresis and purified with the Qiaex II gel purification kit (Qiagen), subsequently individually cloned into the pGemT-Easy vector system (Promega), transformed into the *E. Coli *DH5α strain (Invitrogen), selected and amplified following the manufacturer's instructions. The purification of each plasmidial DNA was carried out as described by Sambrook J et al. [36]. Both DNA strands of each plasmid were sequenced using the BigDye Terminators Kit (Applied Biosystems) on the automated ABI 377 sequencer (Applied Biosystems) and 500 ng of purified plasmid DNA with 3.2 picomoles of either M13 forward or reverse primers as suggested by the manufacturer.

### 5'and 3'RACE experiments

To define the beginning and the end of the mouse CPM mRNA we used the FirstChoice RLM-RACE kit (Ambion) with the Platinum TAQ DNA polymerase (Invitrogen) for the PCRs in the 5'RACE experiments and the Elongase Amplification System (Invitrogen) for the 3'RACE ones. We essentially followed the protocols suggested by the manufacturers, except that in the 5'RACE experiments the RNAs were purified from the CIP reactions using 250 μl of TRIzol (Invitrogen) and the reverse transcriptions were performed with the mouse CPM specific primer pmCPM9r3. Each outer 5'RLM-RACE reaction was done using the primer pmCPMe4r as a 5'RACE gene-specific outer primer and 2 μl of a reverse transcribed previously treated total RNA from 8 day mouse embryos and placenta (EP), 14 day embryos (E) and placenta (P), 3 to 4 month old adult male lungs (L) and adult male peritoneal residing cells (C). Then, 2 μl of these reactions were used as templates in nested inner 5'RLM-RACE reactions with the primer pmCPMe3r as the 5'RACE gene-specific inner primer. Only the outer 3'RLM-RACE reactions were done to define the 3'mouse CPM ends, performed with the primer pmCPMe8f as the 3'RLM-RACE gene specific outer primer and as template the total cDNAs from total RNA of adult mouse lung, and 14 day embryos and placentas. The resulting amplified products of the 5'and 3'RACE reactions were purified after agarose gel electrophoresis using the Qiaex II gel extraction kit (Qiagen), cloned into pGemT-Easy vector (Promega) and sequenced.

### Northern blot

Approximately 30 μg of purified total RNA from the indicated samples were used to prepare a Hybond-N+ nylon membrane (Amersham) blot after electrophoresis in a 1% agarose gel, and hybridized to a phosphorus 32 labelled mouse CPM anti-sense ssDNA probe essentially as described by Brown and colleagues [37], except that we only checked the RNA and Transfer quality by Methylene Blue staining the blot previously to hybridizing, and the mouse CPM probe was prepared by a labelling PCR. This labelling PCR was carried out in a final volume of 50 μl using as template approximately 15 ng of a DNA fragment corresponding to the nucleotide sequence 1153 to 1350 of mouse CPM RefSeq sequence [GenBank: NM_027468], that was obtained from the pGemT-Easy vector (Promega) containing the previously subcloned and sequenced DNA fragment of the RT-PCR with primer pairs pmCPM2f3 + pmCPM9r2, after Eco RI/Himd 3 endonuclease digestion and a 2% agarose gel electrophoresis with Qiaex II Gel Extraction Kit (Qiagen). This PCR was performed with the TAQ recombinant DNA polymerase (Invitrogen) as suggested by the manufacturer except that only the anti-sense primer mCPM9r2 was added at a final concentration of 1 μM, and 1 μl of [γ-32P]dCTP (6000 Ci/mmol, ≥ 10 mCi/ml) (Amersham) was used instead of the suggested 5 mM dCTP. The PCR temperature protocol was of 94°C for 3 min, followed by 40 cycles of 94°C for 15 seconds, 55°C for 30 seconds and 72°C for 30 seconds, and ended at 4°C. The probe was cleaned from unincorporated nucleotides using the ProbeQuant tm G-50 Micro Columns (Amersham Biosciences) as instructed by the manufacturer. The hybridized blot was exposed to a phosphor imaging screen for 24 hours and analyzed by the Cyclone Phosphoimager System (Perkin Elmer).

### Quantitative RT-PCR (qRT-PCR)

All reactions were done in triplicate using 5 μl of the samples' total cDNA diluted 1/50, 10 μl of the Power SYBR Green PCR Master Mix (Applied Biosystems), 1 μl of the transcripts specific primer pairs used in the previously described RT-PCRs, the TBP gene specific primer pair pTPBf + pTBPr or the mouse CPM gene specific primers pair pmCPMf + pmCPMr (all primer mixtures with 10 mM of each primer) in a final volume of 20 μl in the 7000 Sequence Detection System (Applied Biosystems) with a temperature cycling of: 50°C for 2 minutes, 95°C for 10 minutes, 40 cycles of 95°C for 15 seconds and 60°C for 30 seconds followed by the dissociation curve standard protocol. For each reaction a value of Cq (cycle of quantification) was obtained in the exponential phase of the PCRs' kinetic curve.

We first performed qPCR runs in duplicate using as template a serial dilution of the pGemT-Easy vector with each transcript expected amplicom (previously cloned and sequenced) to evaluate the TM of the expected dissociation curve peak and the sensitivity of the reactions. After confirming the expected single peak in the dissociation curve and obtaining a Cq value for each sample's reaction we calculated the average efficiency of the qPCR for each primer pair using the efficiency of each reaction calculated with the LinRegPCR program [38]. We then calculated the relative expression ratio for each transcript in relation to the TBP expression with the equation:

R_mCPM-T/TBP(%) _= [(E_TBP_)^CqTBP^/(E_mCPM-T_)^CqmCPM-T^] × 100;

were "R_mCPM-T/TBP(%)_" is the average relative expression of the analyzed transcript in a sample presented as percentage of the average expression of the TBP gene in that sample, "E_TBP_" is the average efficiency of all the samples' TBP reactions, "CqTBP" is the average Cq value between the triplicated TBP reactions for the specific sample being analyzed, "E_mCPM-T_" is the average efficiency of all the samples' reactions for the mouse CPM *locus *transcript being analyzed and "CqmCPM-T" is the average Cq value between the triplicate reactions of the same mouse CPM *locus *transcript in question for the analyzed sample.

The average Cq values between the specific triplicate reactions had standard deviations lower than the recommended limit of 0.3 cycles, except four values that had standard deviation between 0.3 and 0.5 cycle, but these were Cqs above 30 cycles, where these degrees of standard deviation are acceptable [39-41].

### Statistical analysis

Unless otherwise indicated, the data are expressed as mean ± SEM. The analysis of the Pearson correlation coefficients was done with the GraphPad Prism software (GraphPad Software Incorporated) using the mean value of relative expression shown on table 2.

## Authors' contributions

AOG conceived and designed the study, performed the bioinformatics analysis, prepared all the samples, carried out the molecular biology experiments and drafted the manuscript. FLM participated in the designing of the extra transcripts detection RT-PCR and qPCR and cloning of respective amplicons. VSA participated in the design, execution and analysis of the northern blot experiment. BAC participated in the design and analysis of the northern blot. JBP conceived the study and participated in its design and coordination and helped to draft the manuscript. All authors read and approved the final manuscript.

## Supplementary Material

Additional file 1 **The CAGE-tag sequences analyzed.** Table showing the CAGE transcriptional Start Site identifier (CAGE TSS) and the CAGE tag ID (when available) of the CAGE sequences that support the indicated mouse CPM *locus *transcripts or that maps to the 5'or 3'region of the *locus*. Click here for file

Additional file 2 **The cDNA sequences analyzed to define mouse CPM *locus *transcripts.** Table showing the GenBank Accession numbers of the cDNA sequences that support the indicated mouse CPM *locus *transcripts or maps to the 3' end of the CPM gene. Click here for file

Additional file 3 **The cDNA fragments obtained by RACE 5' to define the mouse CPM start of transcription.** Figure of a negative image of agarose gel electrophoresis of the cDNA fragments obtained by RACE 5' to define the mouse CPM start of transcription, prior to sequencing. Click here for file

Additional file 4 **The genomic region of the mouse CPM gene transcription start.** Figure representing cDNA and CAGE tag position on the nucleotide sequence of the mouse CPM gene start of transcription genomic region. Click here for file

Additional file 5 **The classical polyadenylation signals in the 3' end of the mouse CPM gene genomic sequence.** Figure showing the position of the classical polyadenylation signals in the nucleotide sequence of the 3' end of the mouse CPM gene genomic sequence. Click here for file

Additional file 6 **The deduced sequence for the mouse CPM *locus *transcript 03, 07, 09 and 10.** The nucleotide sequences of the mouse CPM *locus *transcript 03, 07, 09 and 10 deduced from the analysis of the cDNAs attributed to them. Click here for file

Additional file 7 **Primers used in this study.** Table of the primers used in this work and their nucleotide sequence. Click here for file
