# Supplementary material for: Multiple RNAs from the mouse carboxypeptidase M locus: functional RNAs or transcription noise?
Source: BMC Mol Biol. 2009 Feb 8;10:7. doi: 10.1186/1471-2199-10-7 (PMC2644694; doi:10.1186/1471-2199-10-7)
Supplement: Additional file 1 — The CAGE-tag sequences analyzed. Table showing the CAGE transcriptional Start Site identifier (CAGE TSS) and the CAGE tag ID (when available) of the CAGE sequences that support the indicated mouse CPM locus transcripts or that maps to the 5'or 3'region of the locus. [file 1471-2199-10-7-S1.doc]

### Additional file 1

### The CAGEtag sequences analyzed

| **Mouse CPM *locus* transcripts** | **CAGE TSS ^#^** | **CAGE tag ^&^** |
| --- | --- | --- |
| 01 and 02 | T10F06FFD6E7 | 075AC33F1809 |
|  |  | 113BB68H0811 |
|  |  | 087AA43M1701 |
| 04 | T10F0700089B | 080AB08B0107 |
|  |  | 080AB31H1304 |
|  |  | 079AC36F1104 |
|  |  | 101AA40B0905 |
|  |  | 038AB47H1901 |
|  |  | 007AA01P0406 |
|  | T10F07000969 | 077AA07B0907 |
| 06 | T10F07006E16 | 069AD22B1706 |
|  | T10F07006E77 | 069AD03O0909 |
|  |  | 067AC55N0204 |
| 08 | T10F07007594 | 075AC79E0706 |
|  | T10F070076C7 | 069AC92H0508 |
|  |  | 069AF92H0508 |
| 12 | T10F06FFFD55 | 067AB63A0302 |
| 13 | T10F06FFFF81 | 069AF89J0106 |
| 13 | T10F07000043 | 121BA49C0407 |
| 14 | T10F07000541 | 067AB78J1707 |
| 15 | T10F07001053 | 069AB75P1903 |
| 16 | T10F07004DA8 | 003AA44L0513 |
| 17 | T10F07005AC8 | 075AC53N0501 |
|  |  | 085AA21F1301 |
| 19 | T10F070065D6 | 082AC50P1302 |
| 22 | T10F0700AADD | 069AC41E1005 |
| 23 | T10F0700AE0D | 067AA79H1907 |
| 24 | T10F0700AFF1 | 069AA57L0501 |
|  | T10F0700B037 | 067AA73H2203 |
| 25 | T10F0700B490 | Not avalaible |
| 26 | T10R06FFD71D | Not avalaible |
| 29 | T10R07002C61 | Not avalaible |
| 30 | T10R07003F82 | Not avalaible |
| 31 | T10R07005D41 | Not avalaible |
|  | T10R07005D45 | Not avalaible |
|  |  | Not avalaible |
|  |  | Not avalaible |
| 33 | T10R07008EFF | Not avalaible |

# CAGE transcriptional Start Site identifier

^&^ CAGE tag ID.
