# Supplementary material for: Multiple RNAs from the mouse carboxypeptidase M locus: functional RNAs or transcription noise?
Source: BMC Mol Biol. 2009 Feb 8;10:7. doi: 10.1186/1471-2199-10-7 (PMC2644694; doi:10.1186/1471-2199-10-7)
Supplement: Additional file 2 — The cDNA sequences analyzed to define mouse CPM locus transcripts. Table showing the GenBank Accession numbers of the cDNA sequences that support the indicated mouse CPM locus transcripts or maps to the 3' end of the CPM gene. [file 1471-2199-10-7-S2.doc]

**Additional file 2**

**The cDNA sequences analyzed to define mouse CPM *locus*** transcripts.

| **Mouse CPM *locus* Transcript** |  |  | **cDNAs** |  |  |
| --- | --- | --- | --- | --- | --- |
| **01** | [BB605320](http://www.ncbi.nlm.nih.gov/UniGene/seq.cgi?ORG=Mm&SID=8944007) | [BC088735](http://www.ncbi.nlm.nih.gov/UniGene/seq.cgi?ORG=Mm&SID=22708553) | [BY103003](http://www.ncbi.nlm.nih.gov/UniGene/seq.cgi?ORG=Mm&SID=10125707) | [CA556932](http://www.ncbi.nlm.nih.gov/UniGene/seq.cgi?ORG=Mm&SID=9993899) | [CJ167563](http://www.ncbi.nlm.nih.gov/UniGene/seq.cgi?ORG=Mm&SID=27336151) |
|  | [BB618825](http://www.ncbi.nlm.nih.gov/UniGene/seq.cgi?ORG=Mm&SID=9314491) | [BC100404](http://www.ncbi.nlm.nih.gov/UniGene/seq.cgi?ORG=Mm&SID=26596501) | [BY238179](http://www.ncbi.nlm.nih.gov/UniGene/seq.cgi?ORG=Mm&SID=10240883) | [CA561567](http://www.ncbi.nlm.nih.gov/UniGene/seq.cgi?ORG=Mm&SID=9998534) | [CK393561](http://www.ncbi.nlm.nih.gov/UniGene/seq.cgi?ORG=Mm&SID=18258549) |
|  | [BB619932](http://www.ncbi.nlm.nih.gov/UniGene/seq.cgi?ORG=Mm&SID=9315336) | [BI158512](http://www.ncbi.nlm.nih.gov/UniGene/seq.cgi?ORG=Mm&SID=9167550) | [BY253880](http://www.ncbi.nlm.nih.gov/UniGene/seq.cgi?ORG=Mm&SID=10256421) | [CF169680](http://www.ncbi.nlm.nih.gov/UniGene/seq.cgi?ORG=Mm&SID=16472441) | [CN701296](http://www.ncbi.nlm.nih.gov/UniGene/seq.cgi?ORG=Mm&SID=20066949) |
|  | [BB847000](http://www.ncbi.nlm.nih.gov/UniGene/seq.cgi?ORG=Mm&SID=9462905) | [BI653650](http://www.ncbi.nlm.nih.gov/UniGene/seq.cgi?ORG=Mm&SID=9218423) | [BY260250](http://www.ncbi.nlm.nih.gov/UniGene/seq.cgi?ORG=Mm&SID=10262791) | [CF170083](http://www.ncbi.nlm.nih.gov/UniGene/seq.cgi?ORG=Mm&SID=16474409) |  |
|  | [BB854729](http://www.ncbi.nlm.nih.gov/UniGene/seq.cgi?ORG=Mm&SID=9473711) | [BQ560185](http://www.ncbi.nlm.nih.gov/UniGene/seq.cgi?ORG=Mm&SID=9718035) | [BY260794](http://www.ncbi.nlm.nih.gov/UniGene/seq.cgi?ORG=Mm&SID=10263335) | [CF171338](http://www.ncbi.nlm.nih.gov/UniGene/seq.cgi?ORG=Mm&SID=16474980) |  |
|  | [BC047389](http://www.ncbi.nlm.nih.gov/UniGene/seq.cgi?ORG=Mm&SID=14814675) | [BY059442](http://www.ncbi.nlm.nih.gov/UniGene/seq.cgi?ORG=Mm&SID=10082153) | [BY262391](http://www.ncbi.nlm.nih.gov/UniGene/seq.cgi?ORG=Mm&SID=10264932) | [CF172288](http://www.ncbi.nlm.nih.gov/UniGene/seq.cgi?ORG=Mm&SID=16477171) |  |
|  | [BC055073](http://www.ncbi.nlm.nih.gov/UniGene/seq.cgi?ORG=Mm&SID=16444404) | [BY061465](http://www.ncbi.nlm.nih.gov/UniGene/seq.cgi?ORG=Mm&SID=10083847) | [BY745721](http://www.ncbi.nlm.nih.gov/UniGene/seq.cgi?ORG=Mm&SID=10748629) | [CJ161965](http://www.ncbi.nlm.nih.gov/UniGene/seq.cgi?ORG=Mm&SID=27330553) |  |
| **01 and 02** | [BB571313](http://www.ncbi.nlm.nih.gov/UniGene/seq.cgi?ORG=Mm&SID=9002154) | [BB584540](http://www.ncbi.nlm.nih.gov/entrez/viewer.fcgi?db=nucest&id=11481084) | [BY231139](http://www.ncbi.nlm.nih.gov/UniGene/seq.cgi?ORG=Mm&SID=10233843) |  |  |
| **02** | [BP760251](http://www.ncbi.nlm.nih.gov/entrez/viewer.fcgi?db=nucest&val=50218949) | [BP762767](http://www.ncbi.nlm.nih.gov/UniGene/seq.cgi?ORG=Mm&SID=21118781) | [BY083506](http://www.ncbi.nlm.nih.gov/UniGene/seq.cgi?ORG=Mm&SID=10105738) |  |  |
| **03** | [AA218047](http://www.ncbi.nlm.nih.gov/entrez/viewer.fcgi?db=nucest&id=1827032) | [AI390758](http://www.ncbi.nlm.nih.gov/entrez/viewer.fcgi?db=nucest&id=4216765) |  |  |  |
| **05** | [BG413053](http://www.ncbi.nlm.nih.gov/entrez/viewer.fcgi?db=nucest&id=13318606) | [CF587082](http://www.ncbi.nlm.nih.gov/entrez/viewer.fcgi?db=nucest&id=35200344) |  |  |  |
| **06 and 01** | [BB585799](http://www.ncbi.nlm.nih.gov/UniGene/seq.cgi?ORG=Mm&SID=8901593) | [BE985033](http://www.ncbi.nlm.nih.gov/UniGene/seq.cgi?ORG=Mm&SID=8860389) | [BM942769](http://www.ncbi.nlm.nih.gov/UniGene/seq.cgi?ORG=Mm&SID=9657210) |  |  |
| **07** | [AA589379](http://www.ncbi.nlm.nih.gov/UniGene/seq.cgi?ORG=Mm&SID=7339714) | [AK087332](http://www.ncbi.nlm.nih.gov/UniGene/seq.cgi?ORG=Mm&SID=10850116) | [BB664561](http://www.ncbi.nlm.nih.gov/UniGene/seq.cgi?ORG=Mm&SID=9353872) | [DV040261](http://www.ncbi.nlm.nih.gov/UniGene/seq.cgi?ORG=Mm&SID=27414472) |  |
| **08 and 01** | [BX519315](http://www.ncbi.nlm.nih.gov/UniGene/seq.cgi?ORG=Mm&SID=16132882) | [CF724999](http://www.ncbi.nlm.nih.gov/UniGene/seq.cgi?ORG=Mm&SID=17060216) |  |  |  |
| **09** | [CA578066](http://www.ncbi.nlm.nih.gov/entrez/viewer.fcgi?db=nucest&id=25126457) | [CA578083](http://www.ncbi.nlm.nih.gov/entrez/viewer.fcgi?db=nucest&id=25126474) |  |  |  |
| **10** | [AK017670](http://www.ncbi.nlm.nih.gov/UniGene/seq.cgi?ORG=Mm&SID=9074712) | [BY717448](http://www.ncbi.nlm.nih.gov/UniGene/seq.cgi?ORG=Mm&SID=10719989) | [BY717459](http://www.ncbi.nlm.nih.gov/UniGene/seq.cgi?ORG=Mm&SID=10720000) |  |  |
| **10 and 01** | [BI651625](http://www.ncbi.nlm.nih.gov/UniGene/seq.cgi?ORG=Mm&SID=9216398) | [BQ892264](http://www.ncbi.nlm.nih.gov/UniGene/seq.cgi?ORG=Mm&SID=9750570) | [CB173689](http://www.ncbi.nlm.nih.gov/UniGene/seq.cgi?ORG=Mm&SID=17084370) | [CK379143](http://www.ncbi.nlm.nih.gov/entrez/viewer.fcgi?db=nucest&id=40344385) |  |
| **11** | [AK087584](http://www.ncbi.nlm.nih.gov/entrez/viewer.fcgi?db=nuccore&id=26104371) | [BB665814](http://www.ncbi.nlm.nih.gov/entrez/viewer.fcgi?db=nucest&id=16499447) | [BM244961](http://www.ncbi.nlm.nih.gov/entrez/viewer.fcgi?db=nucest&id=31500279) | [BM244984](http://www.ncbi.nlm.nih.gov/entrez/viewer.fcgi?db=nucest&id=31500297) |  |
| **16 and 01** | [BQ830495](http://www.ncbi.nlm.nih.gov/UniGene/seq.cgi?ORG=Mm&SID=9849822) |  |  |  |  |
| **18** | [CR514008](http://www.ncbi.nlm.nih.gov/entrez/viewer.fcgi?db=nucest&id=49905421) |  |  |  |  |
| **20 and 01** | [AW211027](http://www.ncbi.nlm.nih.gov/entrez/viewer.fcgi?db=nucest&id=6516967) | [BB236008](http://www.ncbi.nlm.nih.gov/UniGene/seq.cgi?ORG=Mm&SID=8345178) | [BB243566](http://www.ncbi.nlm.nih.gov/entrez/viewer.fcgi?db=nucest&id=8936312) | [BI648943](http://www.ncbi.nlm.nih.gov/UniGene/seq.cgi?ORG=Mm&SID=9213716) |  |
| **21 and 01** | [BG089317](http://www.ncbi.nlm.nih.gov/UniGene/seq.cgi?ORG=Mm&SID=9042971) | [CD565787](http://www.ncbi.nlm.nih.gov/UniGene/seq.cgi?ORG=Mm&SID=15964360) |  |  |  |
| **27** | [BY591714](http://www.ncbi.nlm.nih.gov/entrez/viewer.fcgi?db=nucest&id=26926896) |  |  |  |  |
| **28** | [BY450198](http://www.ncbi.nlm.nih.gov/UniGene/seq.cgi?ORG=Mm&SID=10452739) |  |  |  |  |
| **32** | [BB535774](http://www.ncbi.nlm.nih.gov/UniGene/seq.cgi?ORG=Mm&SID=8667573) |  |  |  |  |
| **34** | [BB549552](http://www.ncbi.nlm.nih.gov/entrez/viewer.fcgi?db=nucest&id=16447816) |  |  |  |  |
| **3´ CPM end** | [AA038774](http://www.ncbi.nlm.nih.gov/UniGene/seq.cgi?ORG=Mm&SID=7169348) | [AV235408](http://www.ncbi.nlm.nih.gov/entrez/viewer.fcgi?db=nucest&id=6187921) | [BB191161](http://www.ncbi.nlm.nih.gov/UniGene/seq.cgi?ORG=Mm&SID=8300617) | [BI555771](http://www.ncbi.nlm.nih.gov/UniGene/seq.cgi?ORG=Mm&SID=9206646) | [BY423131](http://www.ncbi.nlm.nih.gov/entrez/viewer.fcgi?db=nucest&id=26695291) |
|  | [AA097365](http://www.ncbi.nlm.nih.gov/UniGene/seq.cgi?ORG=Mm&SID=7194885) | [AV235977](http://www.ncbi.nlm.nih.gov/entrez/viewer.fcgi?db=nucest&id=6188490) | [BB194124](http://www.ncbi.nlm.nih.gov/UniGene/seq.cgi?ORG=Mm&SID=8303580) | [BI649309](http://www.ncbi.nlm.nih.gov/UniGene/seq.cgi?ORG=Mm&SID=9214082) | [BY434294](http://www.ncbi.nlm.nih.gov/entrez/viewer.fcgi?db=nucest&id=26715316) |
|  | [AA098323](http://www.ncbi.nlm.nih.gov/UniGene/seq.cgi?ORG=Mm&SID=7195766) | [AV238416](http://www.ncbi.nlm.nih.gov/UniGene/seq.cgi?ORG=Mm&SID=7844250) | [BB229545](http://www.ncbi.nlm.nih.gov/UniGene/seq.cgi?ORG=Mm&SID=8339001) | [BM118486](http://www.ncbi.nlm.nih.gov/UniGene/seq.cgi?ORG=Mm&SID=9464042) | [BY460667](http://www.ncbi.nlm.nih.gov/UniGene/seq.cgi?ORG=Mm&SID=10463208) |
|  | [AA108732](http://www.ncbi.nlm.nih.gov/entrez/viewer.fcgi?db=nucest&id=1660214) | [AV239838](http://www.ncbi.nlm.nih.gov/UniGene/seq.cgi?ORG=Mm&SID=7845666) | [BB231132](http://www.ncbi.nlm.nih.gov/UniGene/seq.cgi?ORG=Mm&SID=8340583) | [BM225944](http://www.ncbi.nlm.nih.gov/UniGene/seq.cgi?ORG=Mm&SID=9620827) | [BY464036](http://www.ncbi.nlm.nih.gov/entrez/viewer.fcgi?db=nucest&id=26766637) |
|  | [AA529949](http://www.ncbi.nlm.nih.gov/UniGene/seq.cgi?ORG=Mm&SID=7323756) | [AV240787](http://www.ncbi.nlm.nih.gov/UniGene/seq.cgi?ORG=Mm&SID=7846613) | [BB284763](http://www.ncbi.nlm.nih.gov/UniGene/seq.cgi?ORG=Mm&SID=8395910) | [BM230045](http://www.ncbi.nlm.nih.gov/UniGene/seq.cgi?ORG=Mm&SID=9624928) | [BY475769](http://www.ncbi.nlm.nih.gov/UniGene/seq.cgi?ORG=Mm&SID=10478310) |
|  | [AA530297](http://www.ncbi.nlm.nih.gov/UniGene/seq.cgi?ORG=Mm&SID=7324104) | [AV328230](http://www.ncbi.nlm.nih.gov/entrez/viewer.fcgi?db=nucest&id=6368282) | [BB536546](http://www.ncbi.nlm.nih.gov/UniGene/seq.cgi?ORG=Mm&SID=8668345) | [BM251129](http://www.ncbi.nlm.nih.gov/UniGene/seq.cgi?ORG=Mm&SID=9589541) | [BY610735](http://www.ncbi.nlm.nih.gov/UniGene/seq.cgi?ORG=Mm&SID=10613276) |
|  | [AA560972](http://www.ncbi.nlm.nih.gov/entrez/viewer.fcgi?db=nucest&id=2332437) | [AV376234](http://www.ncbi.nlm.nih.gov/entrez/viewer.fcgi?db=nucest&id=6423881) | [BB744891](http://www.ncbi.nlm.nih.gov/entrez/viewer.fcgi?db=nucest&id=16147830) | [BM876066](http://www.ncbi.nlm.nih.gov/UniGene/seq.cgi?ORG=Mm&SID=9641537) | [BY615166](http://www.ncbi.nlm.nih.gov/UniGene/seq.cgi?ORG=Mm&SID=10617707) |
|  | [AA647199](http://www.ncbi.nlm.nih.gov/UniGene/seq.cgi?ORG=Mm&SID=7366224) | [AW213683](http://www.ncbi.nlm.nih.gov/entrez/viewer.fcgi?db=nucest&id=6519927) | [BB746126](http://www.ncbi.nlm.nih.gov/UniGene/seq.cgi?ORG=Mm&SID=9404107) | [BP754901](http://www.ncbi.nlm.nih.gov/UniGene/seq.cgi?ORG=Mm&SID=21100798) | [BY622054](http://www.ncbi.nlm.nih.gov/entrez/viewer.fcgi?db=nucest&id=26957236) |
|  | [AA839176](http://www.ncbi.nlm.nih.gov/UniGene/seq.cgi?ORG=Mm&SID=7405155) | [AW490006](http://www.ncbi.nlm.nih.gov/UniGene/seq.cgi?ORG=Mm&SID=8048613) | [BB769474](http://www.ncbi.nlm.nih.gov/entrez/viewer.fcgi?db=nucest&id=16212016) | [BP755201](http://www.ncbi.nlm.nih.gov/UniGene/seq.cgi?ORG=Mm&SID=21101098) | [BY671969](http://www.ncbi.nlm.nih.gov/entrez/viewer.fcgi?db=nucest&id=27054413) |
|  | [AA856136](http://www.ncbi.nlm.nih.gov/UniGene/seq.cgi?ORG=Mm&SID=7410865) | [AW490604](http://www.ncbi.nlm.nih.gov/entrez/viewer.fcgi?db=nucest&id=7060875) | [BB779633](http://www.ncbi.nlm.nih.gov/UniGene/seq.cgi?ORG=Mm&SID=9548875) | [BP756944](http://www.ncbi.nlm.nih.gov/UniGene/seq.cgi?ORG=Mm&SID=21102841) | [BY704559](http://www.ncbi.nlm.nih.gov/UniGene/seq.cgi?ORG=Mm&SID=10707100) |
|  | [AI047732](http://www.ncbi.nlm.nih.gov/UniGene/seq.cgi?ORG=Mm&SID=7456123) | [BB001997](http://www.ncbi.nlm.nih.gov/entrez/viewer.fcgi?db=nucest&id=8091442) | [BB782420](http://www.ncbi.nlm.nih.gov/UniGene/seq.cgi?ORG=Mm&SID=9551662) | [BP767293](http://www.ncbi.nlm.nih.gov/entrez/viewer.fcgi?db=nucest&id=50225991) | [BY712106](http://www.ncbi.nlm.nih.gov/UniGene/seq.cgi?ORG=Mm&SID=10714647) |
|  | [AI465697](http://www.ncbi.nlm.nih.gov/UniGene/seq.cgi?ORG=Mm&SID=7543129) | [BB008087](http://www.ncbi.nlm.nih.gov/entrez/viewer.fcgi?db=nucest&id=8128444) | [BE852513](http://www.ncbi.nlm.nih.gov/entrez/viewer.fcgi?db=nucest&id=10310852) | [BP767555](http://www.ncbi.nlm.nih.gov/UniGene/seq.cgi?ORG=Mm&SID=21119013) | [CB194279](http://www.ncbi.nlm.nih.gov/UniGene/seq.cgi?ORG=Mm&SID=14288107) |
|  | [AI510168](http://www.ncbi.nlm.nih.gov/UniGene/seq.cgi?ORG=Mm&SID=7553259) | [BB009628](http://www.ncbi.nlm.nih.gov/UniGene/seq.cgi?ORG=Mm&SID=8107102) | [BE946252](http://www.ncbi.nlm.nih.gov/UniGene/seq.cgi?ORG=Mm&SID=8843398) | [BP767760](http://www.ncbi.nlm.nih.gov/entrez/viewer.fcgi?db=nucest&id=50226458) | [CB320886](http://www.ncbi.nlm.nih.gov/UniGene/seq.cgi?ORG=Mm&SID=14778512) |
|  | [AI561591](http://www.ncbi.nlm.nih.gov/entrez/viewer.fcgi?db=nucest&id=4512936) | [BB021721](http://www.ncbi.nlm.nih.gov/entrez/viewer.fcgi?db=nucest&id=8194084) | [BF163424](http://www.ncbi.nlm.nih.gov/UniGene/seq.cgi?ORG=Mm&SID=8798047) | [BQ556787](http://www.ncbi.nlm.nih.gov/UniGene/seq.cgi?ORG=Mm&SID=9714640) | [CB321026](http://www.ncbi.nlm.nih.gov/entrez/viewer.fcgi?db=nucest&id=28845261) |
|  | [AI587754](http://www.ncbi.nlm.nih.gov/UniGene/seq.cgi?ORG=Mm&SID=7567592) | [BB083706](http://www.ncbi.nlm.nih.gov/entrez/viewer.fcgi?db=nucest&id=8648942) | [BF785119](http://www.ncbi.nlm.nih.gov/UniGene/seq.cgi?ORG=Mm&SID=9009293) | [BQ556788](http://www.ncbi.nlm.nih.gov/UniGene/seq.cgi?ORG=Mm&SID=9714641) | [CD805033](http://www.ncbi.nlm.nih.gov/UniGene/seq.cgi?ORG=Mm&SID=16184470) |
|  | [AI596756](http://www.ncbi.nlm.nih.gov/UniGene/seq.cgi?ORG=Mm&SID=7853015) | [BB086010](http://www.ncbi.nlm.nih.gov/UniGene/seq.cgi?ORG=Mm&SID=8230609) | [BG094988](http://www.ncbi.nlm.nih.gov/UniGene/seq.cgi?ORG=Mm&SID=9048642) | [BQ560184](http://www.ncbi.nlm.nih.gov/UniGene/seq.cgi?ORG=Mm&SID=9718034) | [CF611715](http://www.ncbi.nlm.nih.gov/UniGene/seq.cgi?ORG=Mm&SID=17009134) |
|  | [AI597093](http://www.ncbi.nlm.nih.gov/UniGene/seq.cgi?ORG=Mm&SID=7853352) | [BB116852](http://www.ncbi.nlm.nih.gov/UniGene/seq.cgi?ORG=Mm&SID=8218280) | [BG095517](http://www.ncbi.nlm.nih.gov/UniGene/seq.cgi?ORG=Mm&SID=9049171) | [BQ565613](http://www.ncbi.nlm.nih.gov/UniGene/seq.cgi?ORG=Mm&SID=9723345) | [CJ299557](http://www.ncbi.nlm.nih.gov/entrez/viewer.fcgi?db=nucest&id=76523752) |
|  | [AI842820](http://www.ncbi.nlm.nih.gov/UniGene/seq.cgi?ORG=Mm&SID=7767435) | [BB122406](http://www.ncbi.nlm.nih.gov/UniGene/seq.cgi?ORG=Mm&SID=8223834) | [BG144476](http://www.ncbi.nlm.nih.gov/UniGene/seq.cgi?ORG=Mm&SID=9054016) | [BQ830773](http://www.ncbi.nlm.nih.gov/UniGene/seq.cgi?ORG=Mm&SID=9850093) | [CJ304740](http://www.ncbi.nlm.nih.gov/UniGene/seq.cgi?ORG=Mm&SID=27524550) |
|  | [AK004327](http://www.ncbi.nlm.nih.gov/UniGene/seq.cgi?ORG=Mm&SID=9084851) | [BB123801](http://www.ncbi.nlm.nih.gov/entrez/viewer.fcgi?db=nucest&id=8776369) | [BG145929](http://www.ncbi.nlm.nih.gov/UniGene/seq.cgi?ORG=Mm&SID=9055469) | [BQ887613](http://www.ncbi.nlm.nih.gov/UniGene/seq.cgi?ORG=Mm&SID=9748859) | [CK129517](http://www.ncbi.nlm.nih.gov/UniGene/seq.cgi?ORG=Mm&SID=17864073) |
|  | [AK013053](http://www.ncbi.nlm.nih.gov/UniGene/seq.cgi?ORG=Mm&SID=9078300) | [BB124830](http://www.ncbi.nlm.nih.gov/UniGene/seq.cgi?ORG=Mm&SID=8234280) | [BG148888](http://www.ncbi.nlm.nih.gov/UniGene/seq.cgi?ORG=Mm&SID=9058428) | [BU755138](http://www.ncbi.nlm.nih.gov/UniGene/seq.cgi?ORG=Mm&SID=9892843) | [CK347016](http://www.ncbi.nlm.nih.gov/UniGene/seq.cgi?ORG=Mm&SID=18214716) |
|  | [AV011399](http://www.ncbi.nlm.nih.gov/UniGene/seq.cgi?ORG=Mm&SID=7619636) | [BB125333](http://www.ncbi.nlm.nih.gov/entrez/viewer.fcgi?db=nucest&id=8779659) | [BG149056](http://www.ncbi.nlm.nih.gov/UniGene/seq.cgi?ORG=Mm&SID=9058596) | [BX523046](http://www.ncbi.nlm.nih.gov/UniGene/seq.cgi?ORG=Mm&SID=16135956) | [CK387141](http://www.ncbi.nlm.nih.gov/UniGene/seq.cgi?ORG=Mm&SID=18252492) |
|  | [AV015593](http://www.ncbi.nlm.nih.gov/entrez/viewer.fcgi?db=nucest&id=4792585) | [BB129394](http://www.ncbi.nlm.nih.gov/UniGene/seq.cgi?ORG=Mm&SID=8238844) | [BG370667](http://www.ncbi.nlm.nih.gov/UniGene/seq.cgi?ORG=Mm&SID=9100957) | [BX632649](http://www.ncbi.nlm.nih.gov/UniGene/seq.cgi?ORG=Mm&SID=16601980) | [CK387572](http://www.ncbi.nlm.nih.gov/UniGene/seq.cgi?ORG=Mm&SID=18252984) |
|  | [AV132025](http://www.ncbi.nlm.nih.gov/entrez/viewer.fcgi?db=nucest&id=5318260) | [BB146064](http://www.ncbi.nlm.nih.gov/UniGene/seq.cgi?ORG=Mm&SID=8255516) | [BI107000](http://www.ncbi.nlm.nih.gov/entrez/viewer.fcgi?db=nucest&id=14557893) | [BY118829](http://www.ncbi.nlm.nih.gov/UniGene/seq.cgi?ORG=Mm&SID=10773921) | [CK389750](http://www.ncbi.nlm.nih.gov/UniGene/seq.cgi?ORG=Mm&SID=18255289) |
|  | [AV141195](http://www.ncbi.nlm.nih.gov/entrez/viewer.fcgi?db=nucest&id=5345190) | [BB150953](http://www.ncbi.nlm.nih.gov/entrez/viewer.fcgi?db=nucest&id=8805890) | [BI151957](http://www.ncbi.nlm.nih.gov/entrez/viewer.fcgi?db=nucest&id=14611958) | [BY414253](http://www.ncbi.nlm.nih.gov/entrez/viewer.fcgi?db=nucest&id=26678761) | [DV043028](http://www.ncbi.nlm.nih.gov/UniGene/seq.cgi?ORG=Mm&SID=27417239) |
|  | [AV235100](http://www.ncbi.nlm.nih.gov/UniGene/seq.cgi?ORG=Mm&SID=7840936) | [BB187423](http://www.ncbi.nlm.nih.gov/UniGene/seq.cgi?ORG=Mm&SID=8296879) | [BI158958](http://www.ncbi.nlm.nih.gov/UniGene/seq.cgi?ORG=Mm&SID=9167996) | [BY418188](http://www.ncbi.nlm.nih.gov/entrez/viewer.fcgi?db=nucest&id=26685734) |  |

The cDNA sequences that support the indicated mouse CPM *locus* transcripts or maps to the 3´ end of the CPM gene are identified by their GenBank Accession numbers*.*
