# Supplementary material for: Multiple RNAs from the mouse carboxypeptidase M locus: functional RNAs or transcription noise?
Source: BMC Mol Biol. 2009 Feb 8;10:7. doi: 10.1186/1471-2199-10-7 (PMC2644694; doi:10.1186/1471-2199-10-7)
Supplement: Additional file 3 — The cDNA fragments obtained by RACE 5' to define the mouse CPM start of transcription. Figure of a negative image of agarose gel electrophoresis of the cDNA fragments obtained by RACE 5' to define the mouse CPM start of transcription, prior to sequencing. [file 1471-2199-10-7-S3.doc]

**Aditional file 3**

**The cDNA fragments obtained by RACE 5´ to define the the mouse CPM start of transcription**


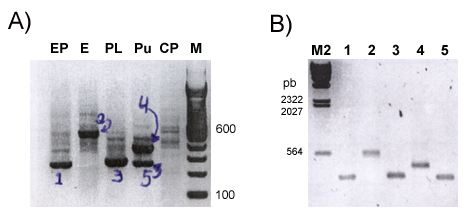
A) Negative image of a 2% agarose gel electrophoresis stained with ethidium bromide and visualized under UV light of the 5´RACE second PCRs of mouse samples: mouse 8 days of gestation embryos and placenta (EP), 14 days of gestation embryos (E), 14 days of gestation placenta (P), 3 to 4 month old adult male lungs (L) and adult male peritoneal residing cells (C). The numbers in blue indicate the gel collected DNA bands that were purified for sequencing. The 600 and 100 base pair DNA bands of the 100 bp DNA ladder (Invitrogen) is also indicated. B) Negative image of a 2% agarose gel electrophoresis stained with ethidium bromide and visualized under UV light of part of the volume of the Qiaex II (Qiagen) purified bands 1 to 5 of the 5´RACE second PCR prior to sequencing. The bands 2 and 4 turned out to be artifacts of the 5´RACE technique. The numbers in the left of the “M2” lane indicate in base pairs the sizes of some of the λDNA/Hind III fragments DNA ladder (Invitrogen) immediately at right.
