# Supplementary material for: Multiple RNAs from the mouse carboxypeptidase M locus: functional RNAs or transcription noise?
Source: BMC Mol Biol. 2009 Feb 8;10:7. doi: 10.1186/1471-2199-10-7 (PMC2644694; doi:10.1186/1471-2199-10-7)
Supplement: Additional file 4 — The genomic region of the mouse CPM gene transcription start. Figure representing cDNA and CAGE tag position on the nucleotide sequence of the mouse CPM gene start of transcription genomic region. [file 1471-2199-10-7-S4.doc]

### Additional file 4

**The genomic region of the mouse CPM gene transcription start**


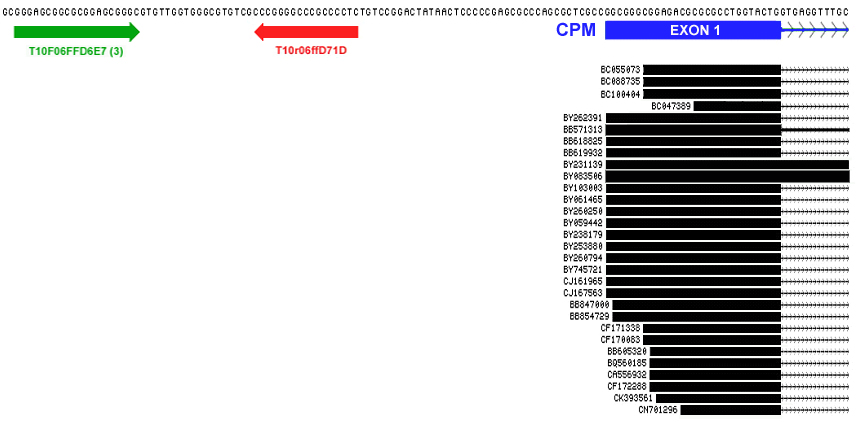


Genomic DNA sequence of the 5´end of the mouse CPM gene showing the position of the CPM exon 1 (blue block), part of CPM intron 1 (arrowed blue horizontal line), the sense CAGEtags (in green), the anti-sense CAGEtag (in red) and the 5´end of the cDNAs that maps to this region identified by their GenBank accession numbers (black blocks).
