# Supplementary material for: Multiple RNAs from the mouse carboxypeptidase M locus: functional RNAs or transcription noise?
Source: BMC Mol Biol. 2009 Feb 8;10:7. doi: 10.1186/1471-2199-10-7 (PMC2644694; doi:10.1186/1471-2199-10-7)
Supplement: Additional file 5 — The classical polyadenylation signals in the 3' end of the mouse CPM gene genomic sequence. Figure showing the position of the classical polyadenylation signals in the nucleotide sequence of the 3' end of the mouse CPM gene genomic sequence. [file 1471-2199-10-7-S5.doc]

**Additional file 5**

**The classical poly-adenilation signals in the 3´ end of the mouse CPM gene genomic sequence**

**5´...GTTACAGTCCCTGGACACGACTCCTACCTCACGAAGCTTACTATTCCAGGGAAATCCCAGCCCTTCAGTGCTCTTAAAAAGGATTTTCACCTCCCGCtgcgatggcagccggattc**

**PA 1**

**catctccgtatccaatccttcgtgcccgatgattccgctgtacaaattcATGCCAAGCCACTCGGCTGCCACAAAGCCTAGTCTGGGCGTGTTTTTCATGACTCTTTTGTACGTATTTTTTAAATAAAGCAAGGTGTGAAACTCGACT**

**TCCGGGAATCAGGGATTGGTTACTCCCGGTTATGGCAACCCTCCCTCCTGTGGGACTGCGATTGGGACAGACTCCACTGTTTTCCTTAAGAAGAAAACCGGATGTTTCCAAACCTGGCCTAGAGCGACCTGTAACGACCAAATCCATCTTCAGTCTGGATAAAGTGGAGGTCACTGCTTAGCTCATGCTGCCCACACAAACGCCACCCCGAAGGAGTCCCAAACACTTGTAAGAAAGTTCAGAAGCAAAACAGATATCCCTACAAGAAAAACAATTTGTCTACGGAGAGCCACGTGGATCAGAGCCGCAAGGACGGGCACTTTCTGGTCACTTGCTGTGTTACGACCTTTCGTGTTATTTGTAGAGAGCTGATATTGTCTATAAGTGGTTCTCCCCAAAGGAAGAAATTTCTAGATAACGGGACTAAGAGCATTTACTCTTGCCAATGGTCTAAGGCCAAATGAGAGAAAATGGACAAACATGTCTGATGTGGGTACAGATGCAGTGGCCTCTGTCTCCCGTGCCTTTGCCCTTGCCCCCTGCACCTGATGCCACAGTCCTACGAAGATGACCCCTCCTGTATAACCCCAGTCCCTTGCCTGAGCCTCAGATGCAACTCCAGCTGCTCACCAGACACACCCACCTGACTACGATGCCTAGGAACTGCAGTTCATCCGGTTGACTGTTGAGACGCCATGCAAGGCTGTTCGATTCCCATATCCAGTCACTGGAAGCTGCTGTATACAGCCTCTCCAGGCCAGTTAGCGTCCCTCCGCCGCTGACCCAGCTGCTGCTCATGGGTATTTTCGCCAAGTCCGTCTTCCACACAGAACCTGGGGAGCTGGGCTGCCCAAAGCTCTCCTGCTCCCTAAACACAACACAGTAAGTGCTCTCGGTCCCAGGTCTGGATTCTGATTGTGTCACGTCTTGTTTCCTGCTCAGTAAAACTCCATTCCATGGGGGGTTAAGAGCACTGACTGCTCTTCCAGAGGTCCTGAGGTCAATTCCCAGCCACCACAT**

**PA2**

**GGTGGCTCACAGCCATCTGTAATGGGATCCGAGGCTGCCTTCTGGTGTGTCTGAAGACAGCAACCATGTAGTCATACACGTAAAATAAATAAATTTTTTAAAAGGAAGAATTCCAAGCATCGTCTTCAACGGGGAAGCCCATGGAGTTCCAGGTGGTCAAGTTCAAGGCCGATCCTTCAAAGAAGTTCCTTTGACTAGTGACTTTACAGCATCGTGGTGTGGAACTGCTCTGTGCTGGCCTTCTCTAGCACGCCATGAGCTCTTTAAGACAAGGATCTGCTTGAGAAATAAT**

**PA3**

**TTCTCAGAGTCTAGCCAAAAAACAGGAAATAAATGGATGGGGAAGTGTCTCAGCTACCGCTACAGACCTTTCCAGAACGCTAAGGATCTGAGTAATTTTGTGTAAATGCGTTTTATGTTTGGCAACATTCAGCTTACTAGTACACACACACACACATACACACTTTTCACGTGTATGTCAAAGAACAAAATCCAAAGATAAATGCAAATGGGTGAGCTGTGAGGCTTGAGGGAAAGGCATTAGCTGTTAGCCAATCTGCCTTCTAGACCAGAAAACAAATTCTTACAGTTCGA**

**AGTAAAGTGTCCCCTGTAAAAGTAACTTTATTCTAACATTAGAAGAAGAGAAAGCTGGGCGTGTTGGCGCACACCTTTAATCCCAACACTCAGAAGGCAGAGGTGGGCGGATGTCTTCGTTTACACAGACAGACCCTGTCTCAAA**

**PA4**

**AGTAAATAAATTAAAAGAAAAAAAAAAGGATAAATATCAAACAGGAACATTAACAGTAACTCCGAGGTGCAGGTTTCTAAGTGACATGCATTTCCCACAGGAAGTAAGAACATGCGGGGTTTGACAGGCGATGAAGGCTTGAGACTCTAAAGAGATACGTGGGCACCCTGGTGCCCTTTCTTGGTGCCAAACCTAATCTATATTTTAATATCAATAGTCTGCATAGCTTTTTAACCGCTTCTAAATTTGGAAATAGCATGAGTATCTTGATCATTTTATTTAAATAAGCATTAACTTATATCCCAGAAGGCGCGTATTAATAGTTCAAAAAGGGATCTGGGTATATTACATTTGAAGTATATATATAAGACGAAGCCAGGTGTGGTGGTGAACTATTATCCCAGCGCTTGGGAGGCTGAGGCAAGATCGACACAGCTCAAGGCTAGCCTGGACTAGCCATCTTAGAAAAGCAAAACCAGTAAGATATGTAAGAGCAAACTTGGTTCGCCATCAGCTGGTGTTCTGAGTTCTGACTGGTTGACCCTTTCCTAAGATTCCGTGAAAGGAAATGAGCTGTTCGCTGAGCTGCTCATGGGCCTAACCGGAGACTTGGTACTGAAACAACTTGCAAGTGCCCGTGTCTTACCAGAGACTGTGCCAGGGCTTTGCACAGGGCCATTGAGCCACAGCTACGTCTTTATTTTGTTACTTTTTGTTCAGACACAGCATTTCACTAAGCTGTGTAGCCTGCACTTGGAACTCATAGTAAGCCAAACTGGCTTTGGATCTGCCTGCCGTAGACGCTGGAGTAGCTATTACAGACCTGGTCCGCTGTGTCCATATTCAGGTTACATTACTTAAATCAGAAATATATATCAAAACCCAGTGTAAAAATCACTAAAACAGGGTGAGGGGTGTAGCTCAGTGACAGTGTACTTAGCATATGCAAAGGCTTGGGTCTAATCTCCATTAACTACCCCCAAGACAAAACATAACCAGGAAATTATTCTGTTGCCCTCTGCCAGGTAGAGAAAGCAGAAGGAATACTTGGGTCTGTAACTGTTTTGAAAGCAATATACCTTACTAAAGGTGACCTGAAAACGGAAATGCTGGTTACCAAAAACCACGTCCTTTTCAAGGAAACTTTCACAAAGTGCCTAGTTTACATGCGTGCCCATTTTTGAATTGAGATGGTAAAATCCTAGCACCCAGGCAGCAGAGGCAGGTGGATCTCTGTGAGTTCAAGGCCAGTCTTTTCTACAGAGTTCTGGGACCGCCAGGGCTACGCAGAAAAACCCCCGTTTCAAACAAGCAAAAACAGTCCCAGTTTTCTCAGACTCTGCCTGTACTAAAATGGTATTAACATCAGATCTGCTTTCCAATGTATGAGCTGAAGCTGGGTTGAGACGTATCTGTTTCTAGGAAAAATATACAAAGCCATATAGCACACTTATTTTCCTATTGCAACAGGAGTTTCATTCTCAGTCTCGGAGGGGGCATCTAGTGAAGGTTTGTGAGCTCAATTCTGCCTTCCTGGTGACCTCAGGTACGGGAGAGAATGATGCTGGATTAGGTGAAGAGCATCGGGTTCTTGGTGCCTACAACAGATTCTTCACCGAGTTTGTTCTAGACTTGCATCACTAATAAGATGCTGTACTGTGTCTCACAGGCCGTTTAATCAAACCTTAAGCTCACAAGCCAACCCACAGCTCTGTGCGAGAGACTTCATGCGGGTTTTTGTTTAACAAGCTTCTAAGTGATTCCTGCCATTAAAAGCACAGAAGCAGAAGCTATTAAAGGTGCCCAGGTTGAGTGGTGGCAGTGCTGGACCACGCCTTTAATCCCAGTACTCCAGAGGCAGAGGCAGAGGGGACTCTGAGTTCAAATCCAGCCTGCCTGGTCT**

**PA5 PA6**

**ACACAGTGAGTTCCAAGACAGCCAGGGCTACACAGAAAAATAAATAAATTCTCAGATTATCTAAGATTACAACGTTTGCTTGGGAGGGACCATGACTACCTCTGAGCTTATTTTATTTACATTATCTGATTATCAATAAAGAATATTCATATAGTGAATGTTGTCTCTTTCCACTACTGTTTTGTTTGCAAACATAACAAAAATCTACAAACTAGAAAAGACTGCTATATGGGGCTAGAAAGATGGCTCAGGGATTAAGAGCACTGACTTCTTCCAGACTCACCTACACTCTCCTCTCTCCACCTCTTTCTTCTTCCTGCCTCAGACCCCCAAACCCCACCTGTCTCTCTTCCACACAGCTACAGGCTGCAGGCATCTTTATTCGCCAATCGGGGATAACTTGGGGGACAAGATTACAGAGCATCATCTGGGTTTAAGTGAGAATTTCCCAGCCCCTGGAGGCAACCAGGGCCAGTATTTAGCAACACCCAAAACCTAACAGTCTCCGGCCTTCCACCTTGTGGTAGCCTCTTGTTTCTGTCATGATGCATACTGCAAGCCAGCTGACCAGCAAGCTTCCAGATGATTCGCCTGTGGGTCTGCCTGTCATCTCTCATGCtgtggttacagatgagtgccaccatatccacggtttctggggatggaatcagggcacaagacaagcactgagccatctcctccacacaagtatcttaactttaagttaaatcgcaaacatttctccgactcaaaagaaaatccattgcaatataatatattgggaaatccctaggcaaaattgcctaaagtatttaaatttgtagctattaggggctggagagatagctgagtggttaagaacactggctgctctcccagaaa**

**PA7**

**ttctgagttcaaatcccagcaactacatggtggctcacaaccacctgtagtgagacttgatgccctcttatagtaagacagctacagtgtacttataataaataaagctttgagccggagcaagcagccagcagaggtcctaaaattcaattcccaacaaccatatgaaggctcacaaccatctgtacagctacagtgtactcatatacataaaatatataaaccttaaaaaaaaaaaaaaaggcggcatgtagacagctgtgtggtcaagagcagcatctgctcttacagaggacagggttaggctcctagcctggaactcgggtcctaaggatctgatgccctcttccggcctcttcaggcactgcatgcatgtagttcaaggcagtgtatgcacacaatacatatacatgtggacgaattattcatacacacaaa**

**PA8**

**ttttaaaaaggtggagagcaatggatcaatttcccagcacccacagggcagctcacacctgtctgtaactccagttccagggtttctgtcaccctcacagacatatagccataacaccaatgaacataaaataaaaatgaattataaaaagatcactgatgggggctgctgagatggctcggtggttaagagcactgactgctcttccaaaggtcctgagttcaactcccagccaccacatggtgggtcacaaccatctgtaatgagatctgacgccctcttctggtgcgt**

**Mdm2**

**ctgaagacagctacagtgtactcacatataatgaacaaatctttaaaaacaaaatcactgacaaaaaaatgtgtaagacaaatctctttcacaattcttCAATAAACCATTTATTTGCAAATAAATA...3´**

The coding sequence of the mouse CPM RefSeq [GeneBank: NM_027468] last exon is shown highlighted in blue followed by the 3´untranslated region highlithed in Yelow. The eight classical poly-A signals (AATAAA) are indicated as PA1 to PA8 in red. The anti-sense final sequence of the Mdm2 RefSeq [GenBank – Gene ID: 17246] is shown highlighted in green and indicated. In white letters highlighted in red are shown the stretches of multiple adenosines that can be targeted by oligo dT primers and generate uncompleted cDNA from longer RNAs containing these sequences.
