# Supplementary material for: Multiple RNAs from the mouse carboxypeptidase M locus: functional RNAs or transcription noise?
Source: BMC Mol Biol. 2009 Feb 8;10:7. doi: 10.1186/1471-2199-10-7 (PMC2644694; doi:10.1186/1471-2199-10-7)
Supplement: Additional file 6 — The deduced sequence for the mouse CPM locus transcript 03, 07, 09 and 10. The nucleotide sequences of the mouse CPM locus transcript 03, 07, 09 and 10 deduced from the analysis of the cDNAs attributed to them. [file 1471-2199-10-7-S6.doc]

### Additional file 6

**The deduced sequence for the mouse CPM *locus* transcript 03, 07, 09 and 10**

**Mouse CPM *locus* transcript 03:**

5´GTCATGTTTCCATTACTGCAGTTATGAATTTTACTTCCTTCCCCACTGCACACTACTTTAATATTTATTTTTAGGAAATATGATTTCAGACAGCTGATGAATTCTCTTGGTTTTAGACTCTGATTATGTGTGTTTTGTTAGTCATTCCCCATGTTGGGACGTTGGAATACTTTCTGATTCTGTTATAAATGATGATATAAATCCATCGATACATATCTTACATACATGCATTAGTGTACATGCATGCTTTGTCTACAGAATCCAAAGAGGGCATCATATCTCCCTGGAACTGAGGTTCCAACAATAGCAGCTTCCGCGTGGGTGCTGGGAACTTGATGTGGGTCCTCTAGAGAAGCAGCCAGTGCTCTTAACCAGTGAGCTGTCTTTCCAG 3´

**Mouse CPM *locus* transcript 07:**

5´TCCAAGAGCCTAAGACGTCTGGCTGTTACAGTGTGGTCCACTGGTGCATTTTGTTCGAACTGCCTCAGGGAGTTTAGAGTTTCTCAGTGATTAATATACACACATATTTGCACAGTTTATAAAGTTGTGGCCTTTGCCAAAAATAAGTAAAATCTCACCACTGGATTTCTTTCCTCCTTAACTTTTCTAGCATGGTTAGGCGCGGTCACGCCAGCCTTCTGTATAAGGTAGCCTGTTTCAGATCTTGGCTGCTGTATAAGGAACAGTGCTAGGCATGGTGACCAGAGCCTTTTCATTAACTGTTGGTTCTGAAATCTGGCAAGTTTGAGAAGGGCAGAGCAGGGTGGGTGTCAATCTCCGGGTCTGAGACAGTCAGCCTGGGGATGTGTGTCTGGGCTCTCAGTGGGCCTGTCACCTGGGTTCTAATTGTTTTATGGAGCCCACACGTGGCTACCAGCCGGTGCCTCTGAGTCTAAAAGGCGCTGTTCCAAGAACACAAAGTCCCAGAACGCAAACAACTGCCCAGCCTTTGCATGTGTCACAGTAGCTAATGTCTCAGCAAAGCAAGTCACACAGTAGGGGACGGGACTTTAGCTATGATTCCTGAGAGGCCTCCAAAGCAATTATCCACTGAACTTGAAGTCACATCTGGGAAACACCTGGGACCTAAGCCTCCAGTCTGTGCCGAGCAAGACCAACCCCCCCCCCCAACACACACACACACACACACACCCCAATAAACCCTACTGTGCTCAGCAAAACCAAGCCCCCACACACCCAAACACCCACACAATAAACTCTGCAAAATCCTTTTCTTTTAAAGGGAAAACTACAATAATTATGACCTGAACAGAAACTTTCCTGATGCCTTTGAGAATAACAATGTAACAAAGCAACCTGAGACTCTGGCAATCATGGAGTGGCTGAAAACGGAAACATTTGTCCTCTCTGCGAATCTCCATGGGGGTGCCCTGGTGGCCAGTTACCCCTTTGATAATGGCGTACAAGGTAAGTGATGTCCATCCCCCACAGCCATCCCCCCCCCCATAGGCCTGAGCTATAAGCACTGCACCTGTGAACTTTGTGGTCCACACTCTTAACTTCTCTCACTGGACTAGAAAAAGCCTAATTTCCCCAACCATGCACAATCTTTCTTGTGATTGGGTTTTTTTTTTTGGGGGGGGGGGCGAGAAAAAGGATAATAACGAATCTCGTGCATGCCAGGGGGCCCTGAAACAGACAGTGTAGCTGAGAGTGCTCTTGAACTTCTGAACCTCTGGTCCTCACAGGCACGCACCATCGGGCAGCTTATGAATACTGAGAATCAAACCCAGGGCTTTGTGCGTCCTAGGCAGGCCCTGTGCAACCTAAGTTATATGGCCAGCTCTTCCTGGAATTCTTAACCAGTGGATGGGAAATGTGGTTGGCCACCATCGGGGAAATCCTACAGCCAGGTGCAGTTTGTATCACAGACTTAGGTTATATGGCTAGAAGAAAATAAAACCAGAATGGTGTATCTTTTTACAATTTTTTTTTTTTGTAATCATGTGAACATTGTTGACTGTTTCTAGCTCTTCTCAATCTGTGAGTCACACACCACCCCTTTGCGGGGTGGGAAATGACCTTTTCACAGGGGTCCCCTAAGACCATCTGCATATCAGATATTTACCTTACGATTCATAACAGTAGCAAAATTACAGTTATGAAGTAGCAATGAAAATAATTTTATGGTTGTGGGGTCGCCACCCACGTGAGGAACTGTGTTAAAGGGTCACAGCTTTAGGAAGGTTGAGAACCGCTGCTTTAGACTCTGTAGCCAGCACTTAGGAGGTGGAGACCAGCCTGTGTACATAGTGAGTTTCAGGACAGCAGGGACTACTTAGAGAGACTGTGTCTCAAG 3´

**Mouse CPM *locus* transcript 09:**

5´ATTATTATTCCTCTAAAACCAGTGTCTCTGATTTTAGTTTAATTAATTTTATAGTTTACAGAAATATTTTTAAAGATCTGCTTTTTTCGTTTTGTTTTTTGTGTATGCGTGTATGTTTGTGTGAGTCTATGCCACATGTGTGCAGGAGACCAAAAGAAAGGGGTCAAATCCCCTGGCCTGGAGCCACCTGATGCGGGTACTGGGCACCGAACTCAAGTTTGCTGGGAAAGTACCAGTATCCGTCTTTCCAGCCCTACAGAAATACTTTAAAAAACAATCATATTCTGCTGTCTTTCCCAGCCTGTTTATCCCACTACCTTTTTGCAGTAGAGTCAATAAACTAAATTTTCCTTTTTGCAATATTTTTAACTTAAAATGTCTTTGAGAACATGAGTGCCCCATTTACACCGTGTCCTGTCCTCCTCTGAACCTCTTCTTAAAAACAGCCTGTACCCACATAAAATGTAGCCTCTACCCTCTAACCACGCCCCTACTCCTTTCTAGTTAGAATGCAATTTAAGGGGGAGGGGTGCTCTTGAAATGTAGGA 3´

**Mouse CPM *locus* transcript10:**

5´CTTCCACCACTGGTCCTTAACAAAAATGAGATGTGCTTGCTTTGCAGGGGGAATGCAAGATTACAACTACATCTGGGCCCAGTGCTTTGAAATTACCCTGGAGCTGTCGTGCTGTAAATATCCTCGCGAGGAGAAGCTGCCGCTCTTTTGGAACGATAACAAAGCCTCTTTGATCGAATATATAAAACAGGTGCACCTA**GG**GGTAAAGGGTCAAGTGTTCGATCAGAGTGGAGCTCCATTACCGAATGTAATCGTGGAAGTCCAAGACAGAAAGCATATCTGCCCGTTTAGAACCAACAAGCTTGGAGAATACTATCTGCTTCTGCTGCCCGGGTCCTACGTGATCAA**TG**TTACAGTCCCTGGACACGACTCCTACCTCACGAAGCTTACTATTCCAGGGAAATCCCAGCCCTTCAGTGCTCTTAAAAAGGATTTTCACCTCCCGCTGCGATGGCAGCCGGATTCCATCTCCGTATCCAATCCTTCGTGCCCGATGATTCCGCTGTACAAATTCATGCCAAGCCACTCGGCTGCCACAAAGCCTAGTCTGGGCGTGTTTTTCATGACTCTTTTGTACGTATTTTTTAAATAAAGCAAGGTGTGAAACTCGACTTCCGGGAATCAGGGATTGGTTACTCCCGGTTATGGCAACCCTCCCTCCTGTGGGACTGCGATTGGGACAGACTCCACTGTTTTCCTTAAGAAGAAAACCGGATGTTTCCAAACCTGGCCTAGAGCGACCTGTAACGACCAAATCCATCTTCAGTCTGGATAAAGTGGAGGTCACTGCTTAGCTCATGCTGCCCACACAAACGCCACCCCGAAGGAGTCCCAAACACTTGTAAGAAAGTTCAGAAGCAAAACAGATATCCCTACAAGAAAAACAATTTGTCTACGGAGAGCCACGTGGATCAGAGCCGCAAGGACGGGCACTTTCTGGTCACTTGCTGTGTTACGACCTTTCGTGTTATTTGTAGAGAGCTGATATTGTCTATAAGTGGTTCTCCCCAAAGGAAGAAATTTCTAGATAACGGGACTAAGAGCATTTACTCTTGCCAATGGTCTAAGGCCAAATGAGAGAAAATGGACAAACATGTCTGATGTGGGTACAGATGCAGTGGCCTCTGTCTCCCGTGCCTTTGCCCTTGCCCCCTGCACCTGATGCCACAGTCCTACGAAGATGACCCCTCCTGTATAACCCCAGTCCCTTGCCTGAGCCTCAGATGCAACTCCAGCTGCTCACCAGACACACCCACCTGACTACGATGCCTAGGAACTGCAGTTCATCCGGTTGACTGTTGAGACGCCATGCAAGGCTGTTCGATTCCCATATCCAGTCACTGGAAGCTGCTGTATACAGCCTCTCCAGGCCAGTTAGCGTCCCTCCGCCGCTGACCCAGCTGCTGCTCATGGGTATTTTCGCCAAGTCCGTCTTCCACACAGAACCTGGGGAGCTGGGCTGCCCAAAGCTCTCCTGCTCCCTAAACACAACACAGTAAGTGCTCTCGGTCCCAGGTCTGGATTCTGATTGTGTCACGTCTTGTTTCCTGCTCAGTAAAACTCCATTCCATGGGGGGTTAAGAGCACTGACTGCTCTTCCAGAGGTCCTGAGGTCAATTCCCAGCCACCACATGGTGGCTCACAGCCATCTGTAATGGGATCCGAGGCTGCCTTCTGGTGTGTCTGAAGACAGCAACCATGTAGTCATACACGTAAAATAAATAAATTTTTTAAAAGGAAGAATTCCAAGCATCGTCTTCAACGGGGAAGCCCATGGAGTTCCAGGTGGTCAAGTTCAAGGCCGATCCTTCAAAGAAGTTCCTTTGACTAGTGACTTTACAGCATCGTGGTGTGGAACTGCTCTGTGCTGGCCTTCTCTAGCACGCCATGAGCTCTTTAAGACAAGGATCTGCTTGAGAAATAATTTCTCTGAGTCTAGCCAAAAAACAGGAAATAAATGGATGGGGAAGTGTCTCAGCTACCGCTACAGACCTTTCCAGAACGCT 3´

The sequences shown in blue represent the common nucleotides between these possible transcripts and the mouse CPM gene.
