# Supplementary material for: Multiple RNAs from the mouse carboxypeptidase M locus: functional RNAs or transcription noise?
Source: BMC Mol Biol. 2009 Feb 8;10:7. doi: 10.1186/1471-2199-10-7 (PMC2644694; doi:10.1186/1471-2199-10-7)
Supplement: Additional file 7 — Primers used in this study. Table of the primers used in this work and their nucleotide sequence. [file 1471-2199-10-7-S7.doc]

**Additional File 7**

**Primers used in this study**

Name Sequence

pmCPM2F2 5´ CCTCGAGTTCCGATATCACCACC 3´

pmCPM2F3 5´ CTAGATCTGGACATGGACCGCGCGCGCC 3´

pmCPM9R2 5´ CCGCGGCCGCACGTACAAAAGAGTCATG 3´

pmCPM9R3 5´ CCGCGGCCGCCGAGTTTCACACCTTGC 3´

pmCPMe8f 5´ CCGAATGTAATCGTGGAAGTCC 3´

pmCPMpa2 5´ ACGTGTATGACTACATGG 3´

pmCPMpa3 5´ TTTTTTGGCTAGACTCTGAG 3´

pmCPMpa4 5´ AGACAGGGTCTGTCTGTG 3´

pmCPMpa5 5´ TAGCCCTGGCTGTCTTGG 3´

pmCPMpa6 5´ GCTCAGAGGTAGTCATGG 3´

pmCPMpa7 5´ AAGTACACTGTAGCTGTC 3´

pmCPMpa8 5´ GTTCATTGGTGTTATGGC 3´

pmCPMf 5´ AAAGGAACACAGAGTCGGG 3´

pmCPMr 5´ GTGGATCCGGGTGCTGTC 3´

pmCPMt03f 5´ AATATGATTTCAGACAGCTGATG 3´

pmCPMt03r 5´ AGAAAGTATTCCAACGTCCC 3´

pmCPMt07f 5´ TGGCAATCATGGAGTGGCTG 3´

pmCPMt07r 5´ GGACATCACTTACCTTGTACGCC 3´

pmCPMt09f 5´ ACCGTGTCCTGTCCTCCTCTG 3´

pmCPMt09r 5´ GCGTGGTTAGAGGGTAGAGGC 3´

pmCPMt10f 5´ AAAATGAGATGTGCTTGCTTTGC 3´

pmCPMt10r 5´ GCTCCACTCTGATCGAACACTTG 3´

pmCPMe4r 5´ GTGGATCCGGGTGCTGTC 3´

pmCPMe3r 5´ CTGCCCCACAACGAGAACCC 3´

pmTBPf 5´ CCCTATCACTCCTGCCACACC 3´

pmTBPr 5´ CGAAGTGCAATGGTCTTTAGGTC 3´

M13 forward 5´ cccagtcacgacgttgtaaaacg 3´

M13 reverse 5´ agcggataacaatttcacacagg 3´
